# Standardization of Fluorescent Reporter Assays in Synthetic Biology across the Visible Light Spectrum

**DOI:** 10.1021/acssynbio.3c00386

**Published:** 2023-11-20

**Authors:** Lien De Wannemaeker, Friederike Mey, Indra Bervoets, Michiel Ver Cruysse, Geoff S. Baldwin, Marjan De Mey

**Affiliations:** † Centre for Synthetic Biology, Ghent University, Coupure links 653, 9000 Ghent, Belgium; ‡ Vrije Universiteit Brussel, Pleinlaan 2, 1050 Brussels, Belgium; § Imperial College London, Sir Alexander Fleming Building, South Kensington, London SW7 2AZ, United Kingdom

**Keywords:** standardization, plate reader assays, optical density, fluorescence, synthetic biology

## Abstract

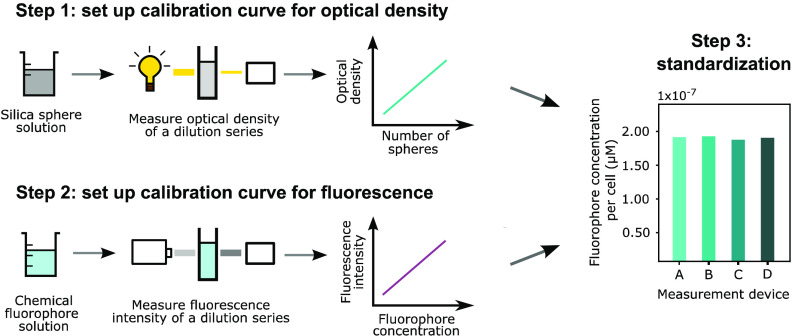

In synthetic biology, Fluorescent reporters are frequently used to characterize the expression levels obtained from both genetic parts such as promoters and ribosome binding sites as well as from complex genetic circuits. To this end, plate readers offer an easy and high-throughput way of characterizing both the growth and fluorescence expression levels of cell cultures. However, despite the similar mode of action used in different devices, their output is not comparable due to intrinsic differences in their setup. Additionally, the generated output is expressed using arbitrary units, limiting reliable comparison of results to measurements taken within one single experiment using one specific plate reader, hampering the transferability of data across different plate readers and laboratories. This article presents an easy and accessible calibration method for transforming the device-specific output into a standardized output expressing the amount of fluorescence per well as a known equivalent fluorophore concentration per cell for fluorescent reporters spanning the visible light spectrum. This calibration method follows a 2-fold approach determining both the estimated number of cells and the equivalent chemical fluorophore concentration per well. It will contribute to the comparison of plate reader experiments between different laboratories across the world and will therefore greatly improve the reliability and exchange of both results and genetic parts between research groups.

## Introduction

Synthetic biology heavily relies on the availability of well-defined and characterized gene expression parts that can be implemented as such or as a part of a more complex genetic circuit. The characterization of these genetic elements is often performed using fluorescent reporters since the expression level of such a reporter is directly correlated to the expression level of the to be characterized genetic part in its specific genetic context and host organism.^1^ Additionally, the generated fluorescent output can easily be measured using, for example, plate reader or flow cytometry assays.

One major drawback to these kinds of measurements is, however, the lack of standardization in the way the generated output is reported in scientific literature.^2−5^ For example, for plate reader assays, where measurements are taken at population level, both the fluorescence and optical density measurements are reported using arbitrary units (a.u.) that are fully dependent on the type and settings of the plate reader used for the measurements. Differences in, for example, the distance between the light source, sample, and detector, the aperture of the detector, as well as the quality and life span of the light source and frequency of maintenance of the plate reader can cause major differences in output between different plate readers.^6^ More specifically for optical density measurements, Stevenson et al. illustrated the importance of cross-calibration between different spectrophotometers, even in the single-scattering regime in order to obtain comparable results.^6^

In addition to the differences that are already created during the measurements, there is no universal protocol on how to report the generated data in a standardized and comparable manner. Beal et al. stressed this issue by comparing the way scientists report the results obtained after cellular fluorescence measurements. A mixture of both normalized and arbitrary units was used, while none of the authors reported results using an independent calibration method.^7^ This lack of standardization in the communication of results causes data to be only comparable within one experiment performed using one single device, rather than across experiments in different laboratories using a variety of plate readers. Therefore, the expression levels generated from the genetic elements that were carefully designed to behave in a standardized manner can be compared only to the parts that were tested within the same experiment. No global conclusion can be drawn based on such data, reducing the reliability of results and therefore hampering the sharing of parts across the synthetic biology community.

**Figure 1:**
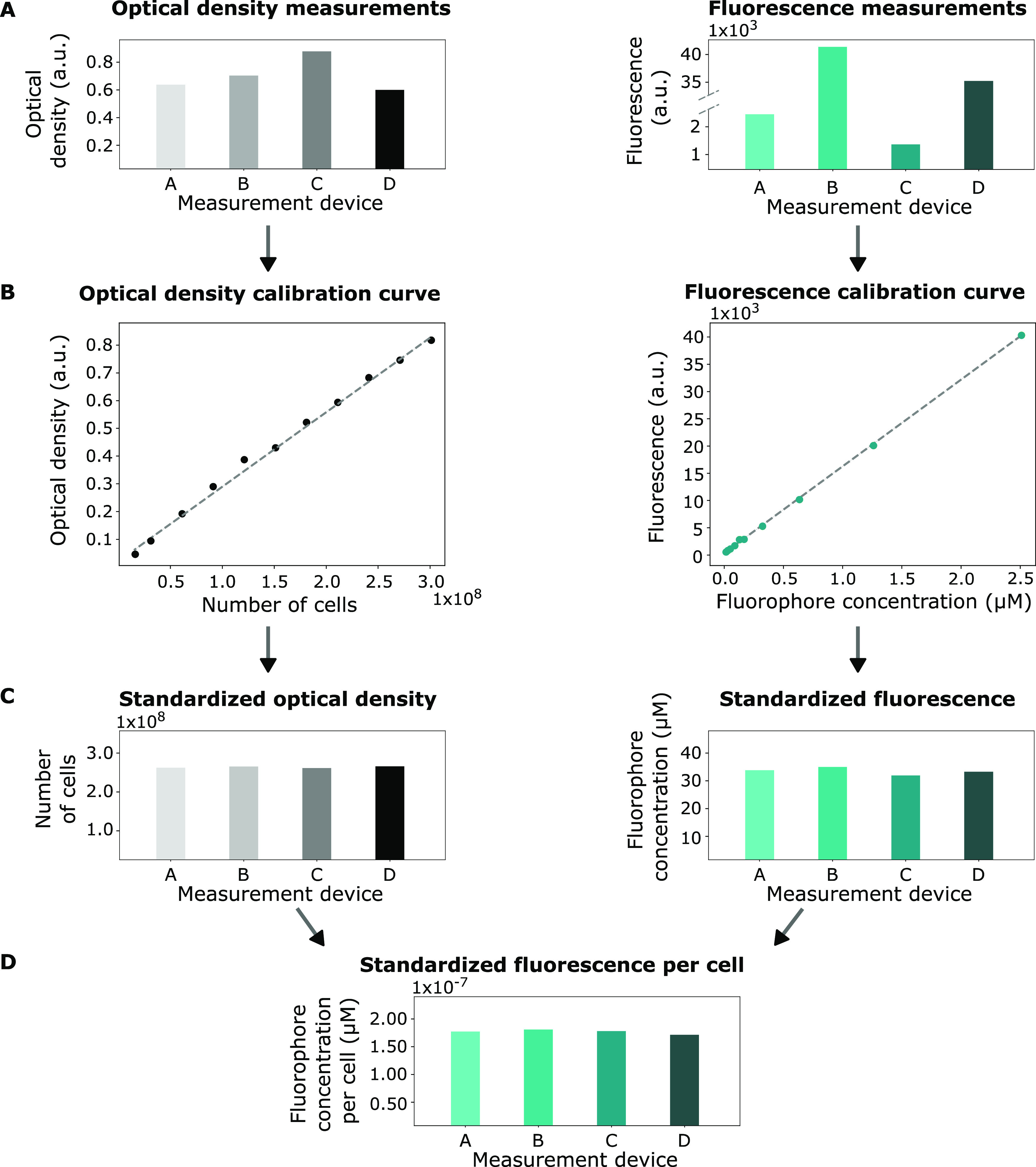
Schematic overview of the calibration workflow used to standardize plate reader fluorescence assays. (A) Output generated by both optical density (left) and fluorescence measurements (right) of the same sample using four different devices. (B) Calibration curves correlating optical density with the exact number of cells (left) and the measured fluorescence to a known fluorophore concentration (right). These standard curves are determined for every device individually. (C) Standardized output of the optical density expressed as the number of cells (left) and fluorescence expressed as a known fluorophore concentration (right). (D) Standardized fluorescence per cell expressed as the fluorophore concentration per cell. a.u. = arbitrary units.

To resolve this issue, a standardized calibration method allowing the conversion of arbitrary units into one universal comparable unit should be introduced. In the past, a calibration protocol in which the output of plate reader measurements of GFP-expressing *Escherichia coli* (*E. coli*) cell cultures was reported as a concentration of the fluorophore sodium fluorescein per cell using silica beads for cell count calibration was developed and evaluated during the iGEM Interlab studies.^4,7,8^ Using two main calibration steps, this method allows transformation of the arbitrary units generated during a plate reader experiment on both the level of optical density and the level of fluorescence expression into standardized equivalent amounts of fluorescence per cell (Figure 1). This powerful method has been proven to be comparable to results obtained from flow cytometry measurements.^8^ More recently, Csibra et al. developed another calibration method, FPCountR, in which purified fluorescent proteins are used for calibration purposes.^9^ While this method was the first to expand the calibration range across the visible light spectrum, it requires more labor-intensive protein purification steps and relies on a conversion factor to obtain the equivalent intracellular fluorescent protein concentrations.

To resolve the limitations of the current standardization methods, we optimized the calibration method for optical density measurements and expanded the equivalent chemical fluorophore portfolio for fluorescent proteins with a wide range of excitation and emission maxima. Doing so, we obtained a standardization method that allowed the data to be represented as an equivalent fluorophore concentration per cell. First, three different easy and accessible cell count methods, namely, microscopic cell counting using a hemocytometer, cell dry weight measurements, and optical density measurements of a known concentration of 1.05 μm silica spheres, were assessed for both their trueness and precision compared to flow cytometry measurements. Second, six different fluorophores with excitation and emission spectra closely resembling those of their respective fluorescent proteins, mKate2, mCherry, OFP, sYFP2, sfGFP, and mTagBFP, were selected and characterized. Finally, the newly developed standardization protocol was assessed by characterization of a naringenin-inducible promoter on four different plate readers using the six selected fluorescent proteins.

## Results and Discussion

### Correlating the Number of Cells to the Optical Density

#### Selection of the Optimal Optical Density Calibration Method

**Figure 2:**
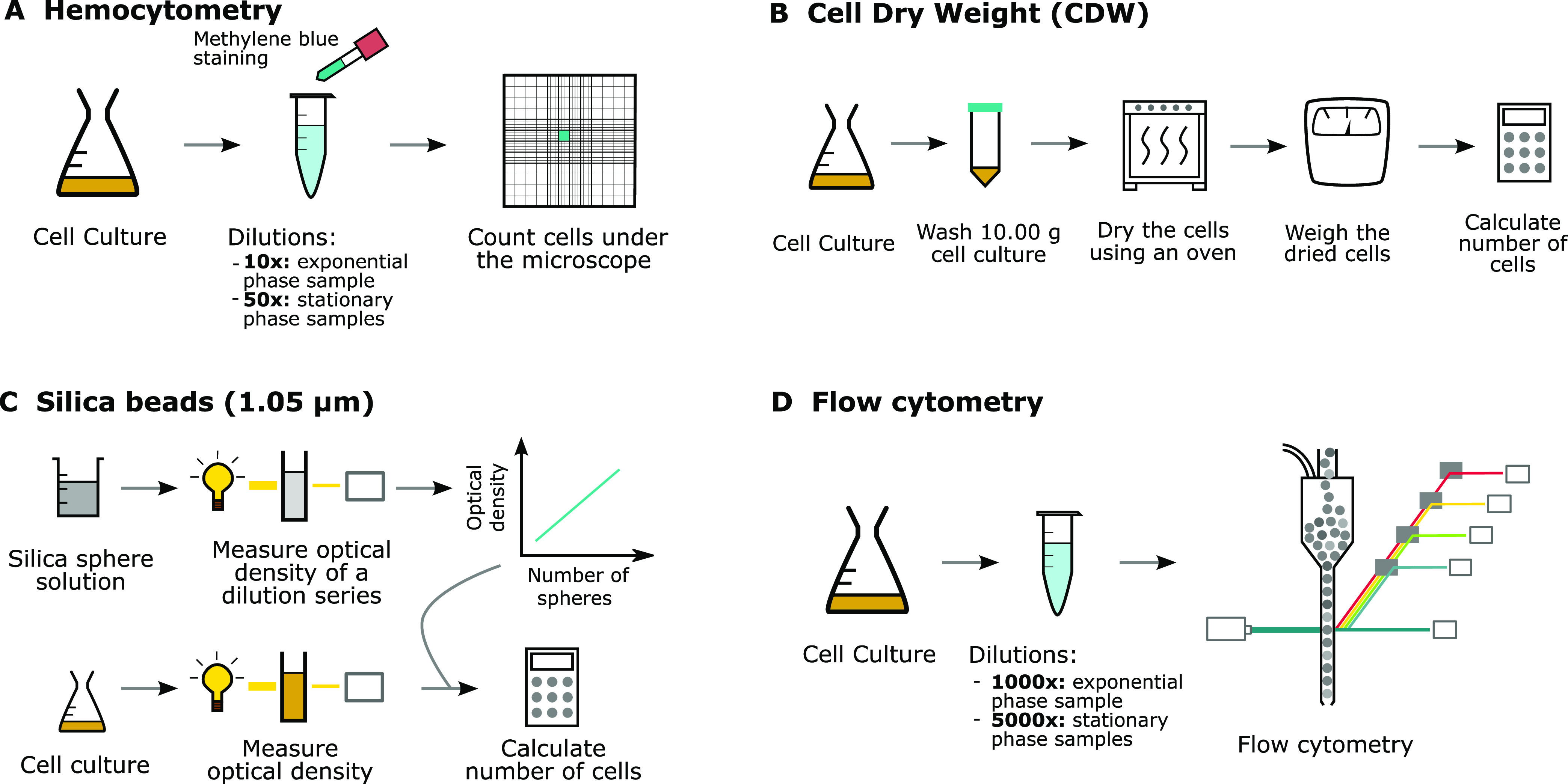
Overview of the four different cell count methods compared during this study. (A) Hemocytometry: the cells are counted under the microscope using a counting chamber. (B) Cell dry weight (CDW) measurements: a known amount of liquid culture broth is washed and dried in an oven. After 2 days, the weight of the dried cells is determined. This weight can be used to determine the number of cells in the original sample. (C) Silica spheres: the number of cells in the culture is determined by using a silica sphere solution with a known concentration of spheres. The used spheres reflect the light in a similar manner to *E. coli*. (D) The cells in the sample are counted using a flow cytometer.

The first step in the standardization protocol consists of a method that calibrates the optical density generated by a specific plate reader using a sample with a known number of cells. To ensure the reliability and widespread use of the protocol, we set the focus on selecting a method with both high accuracy and ease of use. Four frequently used cell count techniques, namely, hemocytometry, cell dry weight (CDW) determination, calibration using silica beads, and flow cytometry, were evaluated in this work (Figure 2).

Since flow cytometry is known to be the most reliable method to determine the amount of cells in a sample, it is used to set the reference value in this work.^8,10^ However, since it is an expensive high-end method not readily available to every lab, it will not be suggested as the standard method for optical density measurement calibration. All cell count techniques were performed using the exact same growth cultures to allow for a reliable comparison of the obtained results between the different methods.

**Figure 3:**
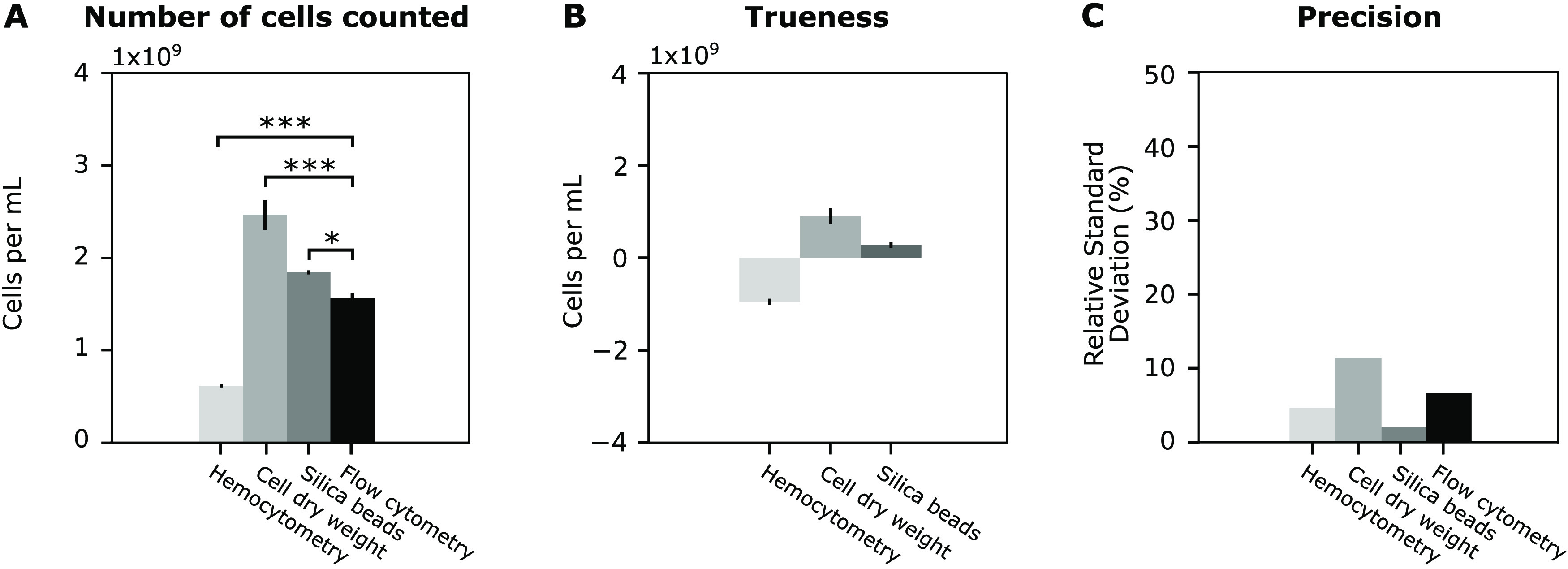
Results obtained for the different cell count methods (hemocytometry, cell dry weight (CDW) determination, silica bead calibration, and flow cytometry) on *E. coli* cell cultures in exponential growth phase. (A) The number of cells per mL obtained by counting the cells using the different methods. Two technical and three biological replicates were measured for each method and growth phase; * indicates *p*-value <0.05, *** indicates *p*-value <0.001 obtained by performing a one-way ANOVA test comparing hemocytometry, CDW determination, and calibration using silica spheres to flow cytometry for each growth phase. (B) Trueness of the cell counts obtained via hemocytometry, CDW determination, and calibration with silica beads using the results obtained with flow cytometry as a reference value. (C) Precision of the different methods. The relative standard deviation is the standard deviation divided by the average amount of cells counted.

Figure 3 shows the cell count and accuracy consisting of both the trueness and precision of the methods tested in this study.^11^ Since flow cytometry is the most reliable method for cell counting, the number of cells obtained using this method is set as the reference value to determine the trueness of the different techniques.^8,10^ The precision, on the other hand, is reported as the relative standard deviation to normalize for the differences in cell counts obtained for the different methods.

The first method, hemocytometry, suffers from a systematic underestimation of the number of cells, which can be explained by the coverslips that are susceptible to the capillary force from the liquid loaded in the counting chamber. These coverslips were used because the ones suitable for hemocytometry resulted in a dark and blurry image at the magnification required to count *E. coli* cells.

Counting cells using CDW determination resulted in an improved estimation of the actual number of cells and improved precision compared to hemocytometry. One drawback of this method is that the weight of the dried cells needs to be converted to an amount of cells using the estimated CDW per cell available in the literature, namely, 2.8 × 10^–13^ g CDW per cell.^12^ This value is determined based on an *E. coli* cell culture in the exponential growth phase in medium containing glucose. A deviation in the actual dry weight per cell caused by deviating conditions, such as the growth phase, medium, or stress levels, may therefore introduce a systematic error. Additionally, depending on the operator, a higher number of cells may be discarded during the washing step, resulting in an underestimation of the actual number of cells, or the supernatant might not be removed completely, causing additional weight due to leftover salts.

The third and last calibration method correlates a known concentration of silica spheres with a diameter of 1.05 μm to the optical density output of a specific plate reader. These spheres or beads were selected since they have a similar size and scatter the irradiated light at a similar angle compared to *E. coli* cells.^6−8^ Based on the results shown in Figure 3, both the trueness and precision of this method are the best of the three proposed methods. However, despite these promising results, several elements can reduce the accuracy of this technique. First, similar to the CDW method, the growth phase, medium, and stress level will have an impact on the way *E. coli* reflects light. Additionally, since the silica spheres sediment rapidly, the solution should be mixed thoroughly just before executing the measurement. Despite these remarks, this method is the best-suited method to correlate the optical density generated by a plate reader to an exact number of cells. Moreover, it has already been extensively tested during different iGEM InterLab studies, in which its potential to successfully calibrate optical densities between different plate readers across the world has been proven.^4,7,8^ Additionally, the silica bead method scores the highest for ease of use, which increases its chances of actually being implemented in a real-life lab setting. Therefore, this method is the recommended calibration technique for optical density measurements of *E. coli*.

#### Determining the Correlation between the Optical Density and the Amount of Silica Beads for the Different Devices

**Figure 4:**
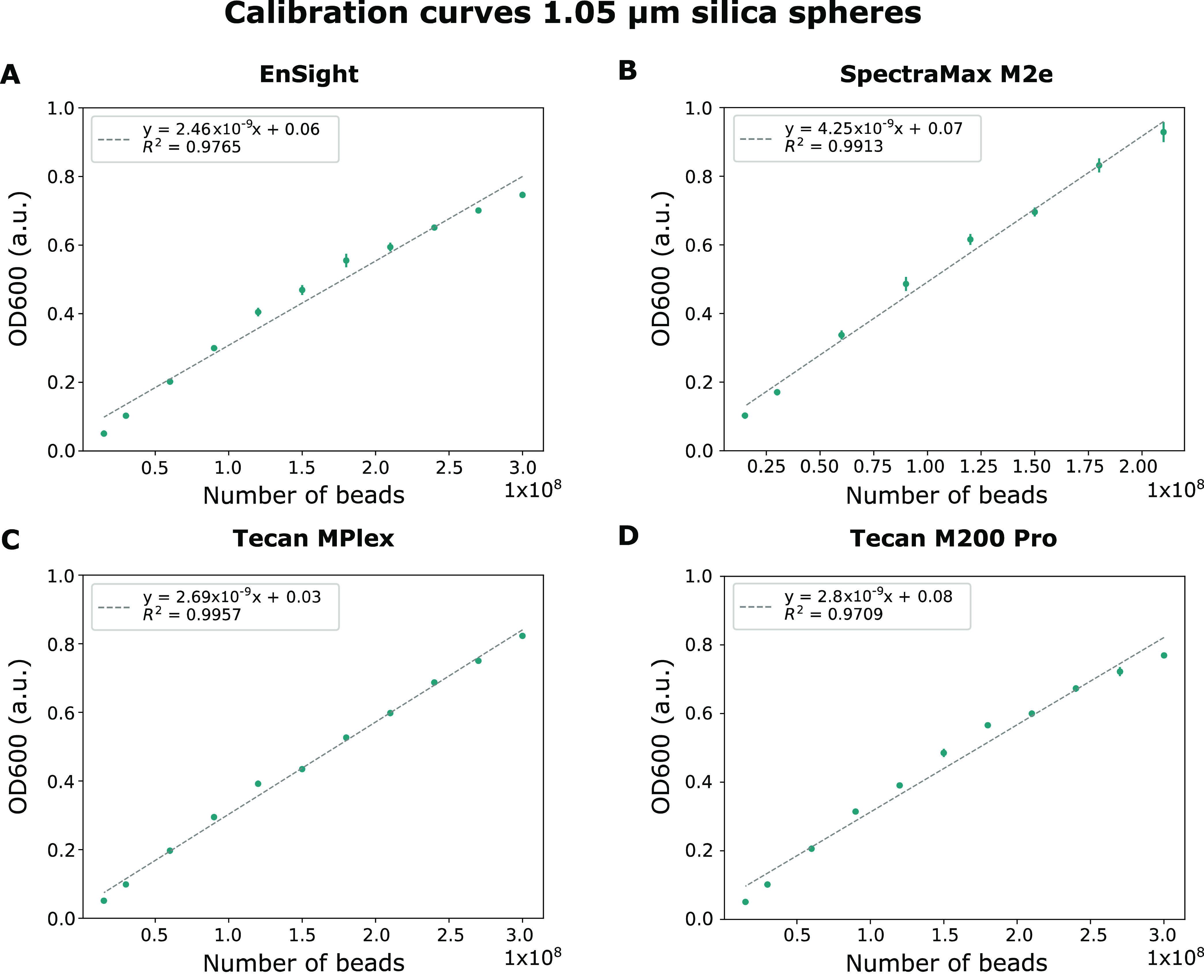
Calibration curves of silica spheres with a diameter of 1.05 μm measured using four different plate readers: (A) EnSight, (B) SpectraMax M2e, (C) Tecan MPlex, and (D) Tecan M200 Pro. OD600 = optical density measured at 600 nm. Error bars are standard errors and *n* = 8. a.u. = arbitrary units.

To determine the correlation between the optical density and the number of beads present in a well, the optical density of a dilution series of a silica sphere solution with a known concentration was measured. Only optical density values smaller than or equal to 1 are taken into account since they fall within the linear range of optical density measurements.^6^ The calibration curves were determined for four different devices, namely, the EnSight, SpectraMax M2e, Tecan MPlex and Tecan M200 Pro plate readers (Figure 4). From these calibration curves, it can already be seen that different optical density values are obtained for the same number of beads per well. The difference is especially remarkable for the SpectraMax M2e plate reader, where the measured optical density is approximately 1.5 times higher compared to the output generated by the other plate readers. This might be due to the life span of the light source and detector of this plate reader since this device is by far the oldest of the tested devices. Generally, this could mean that, with time, the intensity of the light source gradually decreases, which in turn reduces the amount of light that reaches the less sensitive detector and thus increases the optical density value that is reported by the device. This phenomenon narrows down the operational range of the plate reader since a lower number of cells will already result in an optical density of 1 when compared to a more recent plate reader.

### Calibration of Fluorescence Measurements

#### Selection of the Appropriate Fluorophores for Standardization

The second part of the proposed standardization protocol consists of the calibration of the fluorescence intensity measured across the visible light spectrum by using different plate readers. To this end, one appropriate chemical fluorophore was chosen for each of the selected fluorescent proteins, mKate2, mCherry, OFP, sYFP2, sfGFP, and mTagBFP, based on their spectral properties, chemical stability, ease of use, and accessibility. The spectral properties of the fluorophore are primordial since the spectrum of the chemical fluorophore should mimic the excitation and emission spectra of the fluorescent protein to allow successful standardization. Next to the spectrum of the fluorophores, their stability in solution is also highly important, since an unstable fluorophore will quickly suffer from reduced fluorescence intensity, which will result in deviations from the true calibration curve if the measurements are not taken immediately after dissolving the fluorophore. Another important element in improving the quality of the calibration protocols is the ease of use of the fluorophores. The ability to dissolve the fluorophores in a solvent with a viscosity comparable to that of water is, for example, highly important to minimize pipetting errors. Additionally, since this calibration protocol only has the desired impact if it is used in all laboratories performing plate reader assays, the fluorophores were chosen to be easily accessible and affordable for all laboratories.

**Figure 5:**
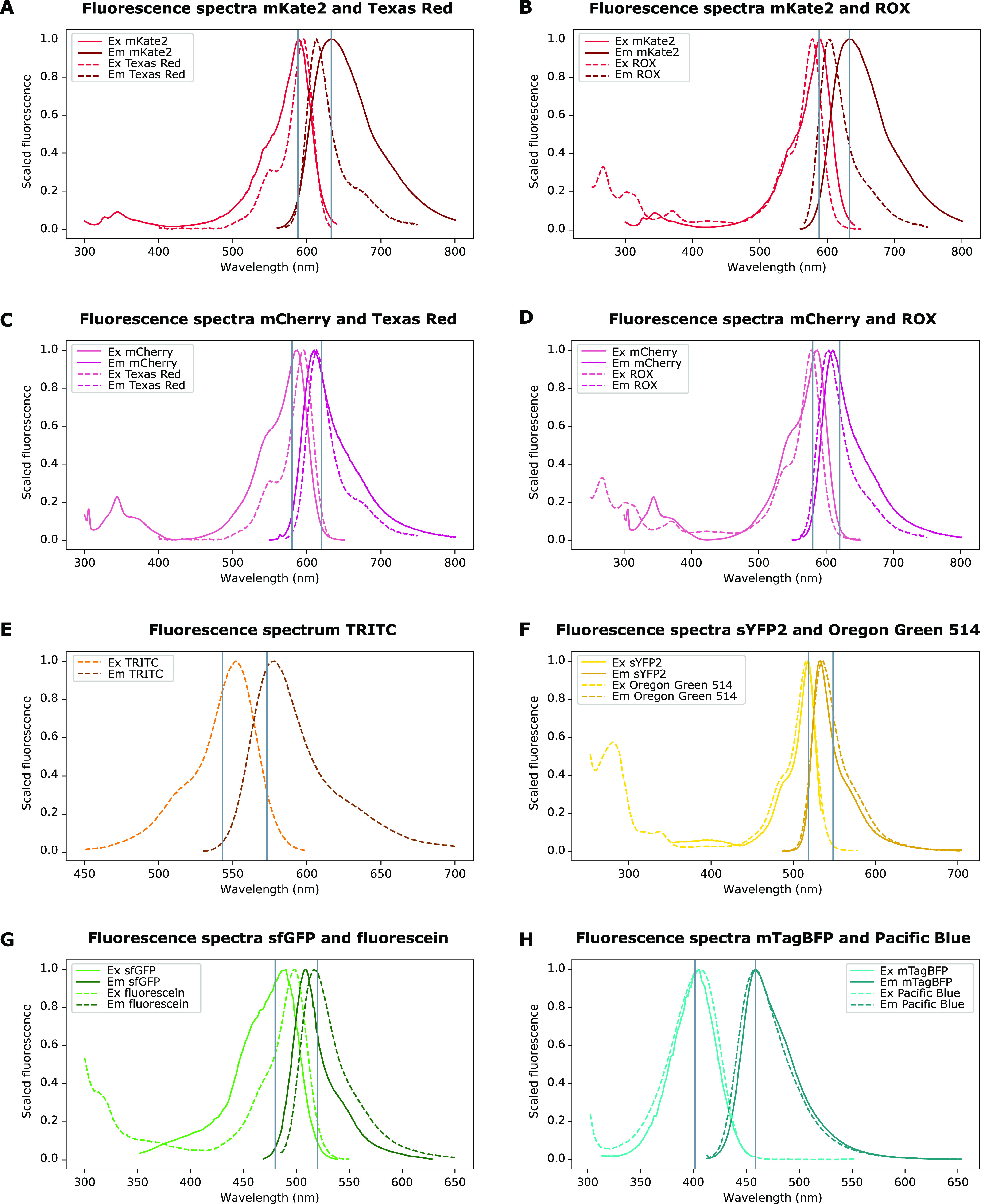
Overview of the excitation and emission spectra of the different fluorescent reporters and their selected cognate chemical fluorophore. Overlay plots of the fluorescence spectra of (A) mKate2 and Texas Red (sulforhodamine 101 chloride), (B) mKate2 and ROX (carboxy-X-rhodamine), (C) mCherry and Texas Red, (D) mCherry and ROX, (E) TRITC (tetramethylrhodamine), (F) sYFP2 and Oregon Green 514, (G) sfGFP and fluorescein, and (H) mTagBFP and Pacific Blue. The scaled fluorescence is the fluorescence rescaled to a maximum value of 1. All spectra were retrieved from fpbase.org.^13^ The fluorescence spectrum of OFP is not yet available on fpbase.org. The vertical lines indicate the excitation (left) and emission (right) wavelengths used during all plate reader experiments.

Two fluorophores, namely, sulforhodamine 101 acid chloride (Texas Red) and carboxy-X-rhodamine (ROX), were selected to serve as equivalent chemical fluorophores for the two fluorescent proteins emitting in the red part of the visible spectrum, i.e., mKate2 and mCherry. Tetramethylrhodamine (TRITC), Oregon Green 514, sodium fluorescein, and Pacific Blue succinimidyl ester were selected for the calibration of the fluorescent proteins OFP, sYFP, sfGFP, and mTagBFP, respectively. Sodium fluorescein was already extensively tested for its suitability as an equivalent fluorophore for GFP expression during the iGEM Interlab studies.^7,8^ This fluorophore was incorporated in this study as an extra validation of both the already established method and the results generated in this study. The absorbance spectra of all fluorophore solutions were characterized (Figure S1), and the molar attenuation coefficients (ε) at the specific maximal absorption wavelength (λ_max_) were determined using the Beer–Lambert law (Table S1). Overlay plots demonstrating the similarity between the excitation and emission spectra of the different fluorescent proteins and the selected fluorophores are shown in Figure 5.^13^ To assess the stability of the selected fluorophores, their fluorescence intensity on the day the stock solutions were made was compared to the intensity obtained after 14 days of storage at −20 °C (Figure S2). Only ROX and fluorescein remain stable after 2 weeks of storage at −20 °C. Texas Red, TRITC, and Oregon Green 514 show a significant decrease in fluorescence intensity after 14 days of storage. This decrease is especially obvious for TRITC. The fluorescence intensity of Pacific Blue, on the other hand, increased after 14 days. To date, there is no explanation for this observation.

#### Calibrating the Fluorescence Output to a Known Concentration of Equivalent Fluorophore

**Figure 6:**
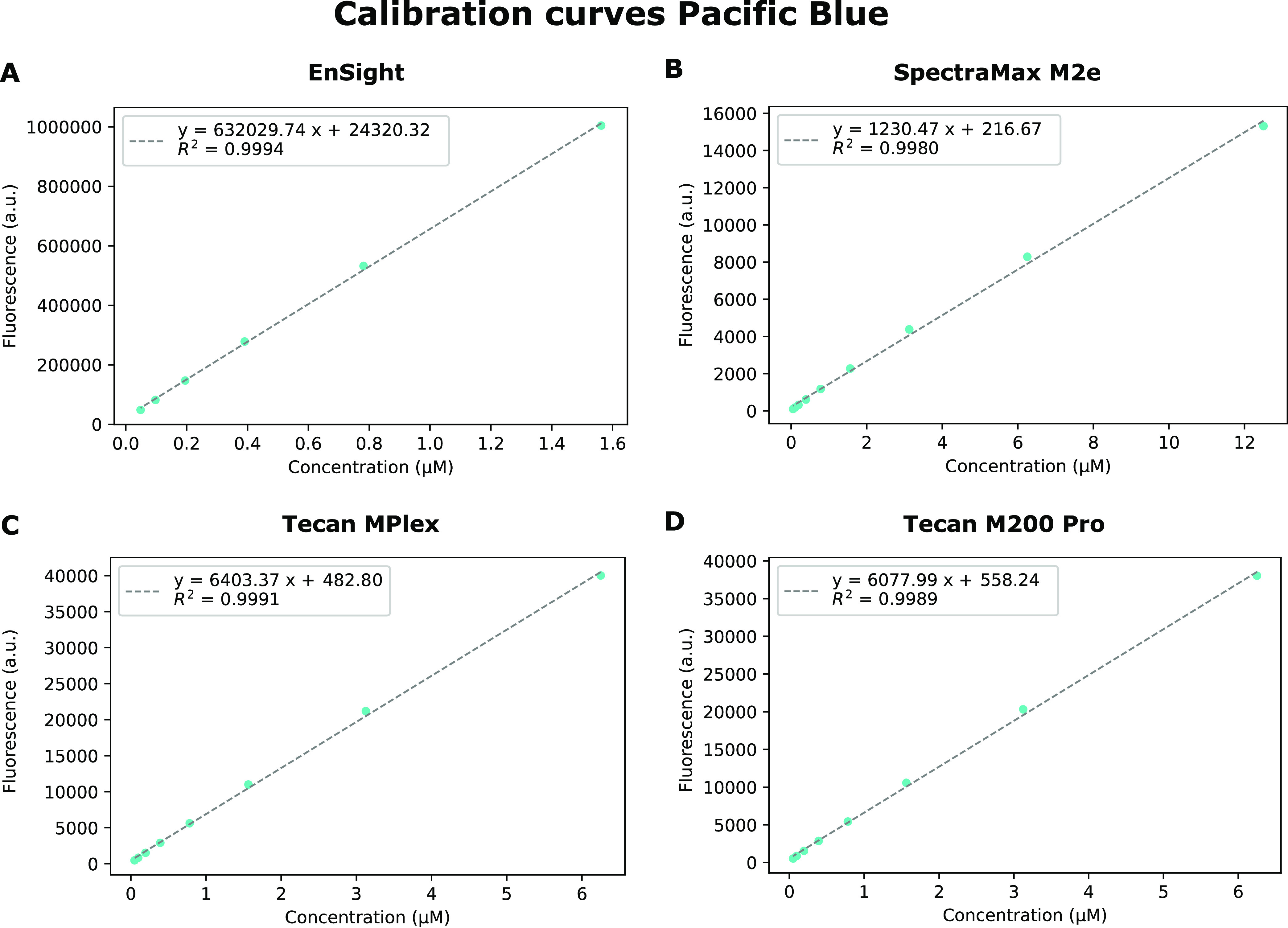
Calibration curves of Pacific Blue measured at four different plate readers: (A) EnSight, (B) SpectraMax M2e, (C) Tecan MPlex, and (D) Tecan M200 Pro. The measurements were taken at the optimal excitation and emission wavelengths for the blue fluorescent protein mTagBFP (399 and 456 nm, respectively). Error bars are standard errors and *n* = 8. a.u. = arbitrary units. The standard curves for Texas Red, ROX, TRITC, Oregon Green 514, and fluorescein can be found in Figures S5–S11.

Next, the output signal of each plate reader was correlated to a known concentration of the chemical fluorophore. All devices used in this study are monochromators capable of measuring across the entire visible light spectrum. Both the Tecan MPlex and Tecan M200 Pro devices allowed manual setting of the gain, while the signal given by the EnSight and SpectraMax M2e plate readers is normalized using a reference signal. The manual gain setting allows setting the optimal gain so that the fluorescent signal of the samples, in this case, the *E. coli* cell cultures expressing the different fluorescent reporters, is situated nicely within the operating range of the detector. Since the optimal gain setting of the cell culture is not necessarily optimal for the measurements of the chemical fluorophore concentrations that are part of the dilution series, only data points within the linear range of the detector were withheld by excluding all values equal to or exceeding the detector limit from the correlation curve. The same principle applied to the devices that did not allow setting the gain value, although the decision making was less straightforward than cutting off the data set at the detector limit. Measuring the calibration curves on both the EnSight and SpectraMax M2e plate readers revealed that in reality the linear range of the detector ends before the detector saturates (Figures S3–S4). To determine which fluorophore concentrations fell outside the linear range, the residuals of the linear regression were assessed. As can be seen in Figure S3F–H, the residuals do not randomly scatter across zero but instead show a strong deviation, indicating that the linear range of the detector is exceeded for the higher fluorophore concentrations. This can be solved either by discarding the data points that fall outside the linear range of the detector or by fitting an exponential curve to the data. The first option was selected since this allows the selection of a linear fit for any device, regardless of whether it allows gain setting or not, which increases the standardization of this calibration method. Figure 6 shows the finalized calibration curves set up using Pacific Blue for the four different plate readers. The standard curves for Texas Red, ROX, TRITC, Oregon Green 514, and fluorescein can be found in Figures S5–S11.

### Validation of the Standardization Protocol

**Figure 7:**
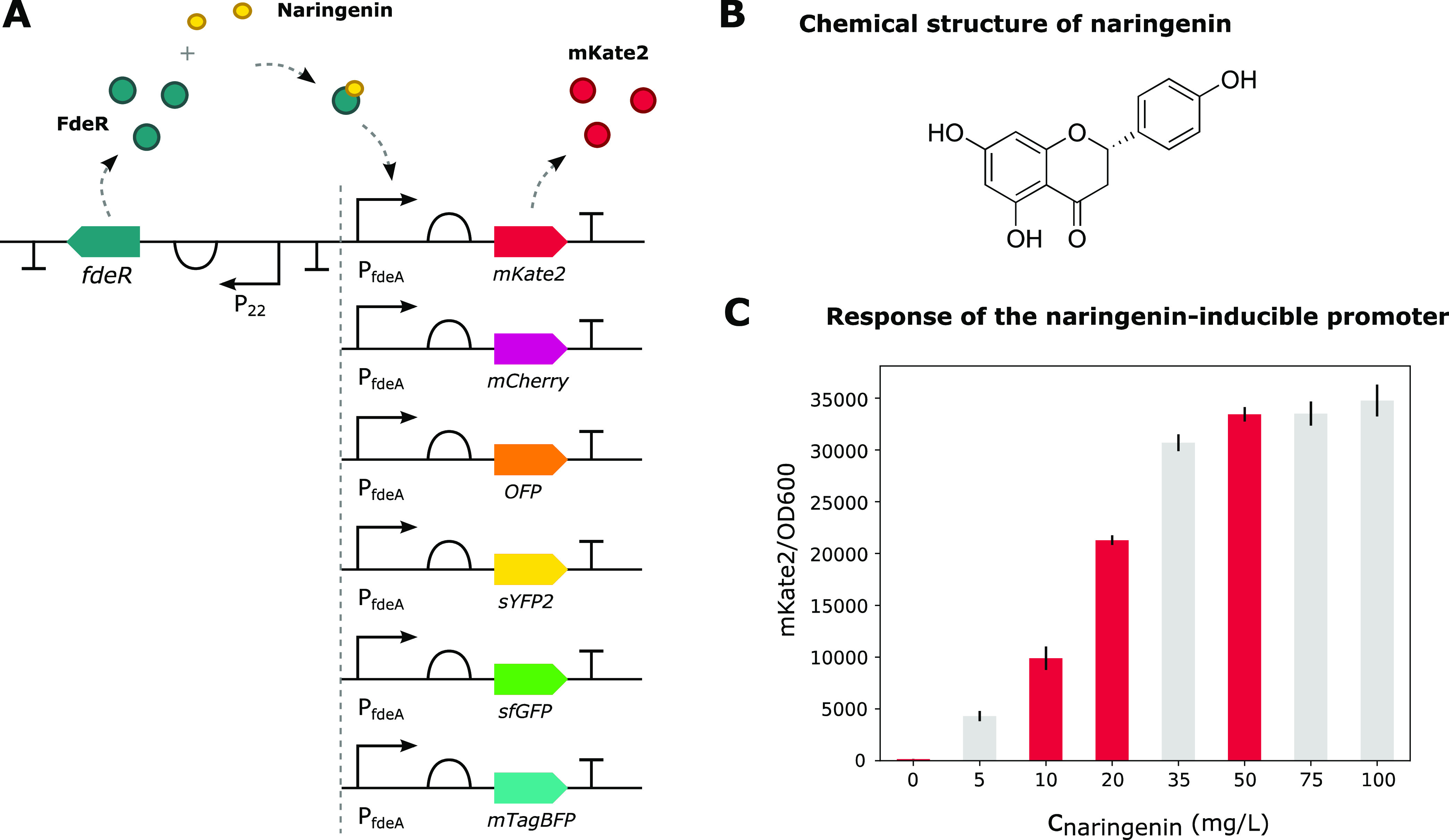
(A) Overview of the different plasmids constructed to validate the standardization protocol for fluorescent reporter assays developed during this work. The different fluorescent reporters are expressed using a naringenin-inducible promoter, allowing easy regulation of the expression level based on the amount of inducer added. Upon addition of the inducer, naringenin, transcription initiation of the downstream gene is enabled. (B) Chemical structure of naringenin. (C) Expression levels generated by the naringenin-inducible promoter on the pBBR1-MCS2 backbone upon addition of different concentrations of inducer. The fluorescent protein mKate2 was used as a reporter molecule. The concentrations 0, 10, 20, and 50 mg/L were selected to obtain expression levels spanning the dynamic range of the naringenin-inducible promoter.

To verify whether the calibration methods for both the optical density and fluorescence measurements result in a comparable output regardless of the device used for the measurements, one single 96-well plate experiment in which the six different fluorescent reporters were expressed by *E. coli* was measured in the four different plate readers. To obtain a range of expression levels of the different proteins, they were cloned downstream of a naringenin-inducible promoter with low leaky expression (Figure 7).^14−16^ Four inducer concentrations, namely, 0, 10, 20, and 50 mg/L, were selected to obtain fluorescence levels spanning the entire dynamic range of the naringenin-inducible promoter (Figure 7C). Doing so, the functionality of the calibration method can be evaluated for low, medium, and high fluorescence intensities. Measurements were taken on the exact same 96-well plate at the four different plate readers during the exponential growth phase. This was done to avoid interexperiment variability. Cell growth was arrested by storing the 96-well plate on ice between measurements.

#### Standardizing Optical Density Measurements between the Different Plate Readers

**Figure 8:**
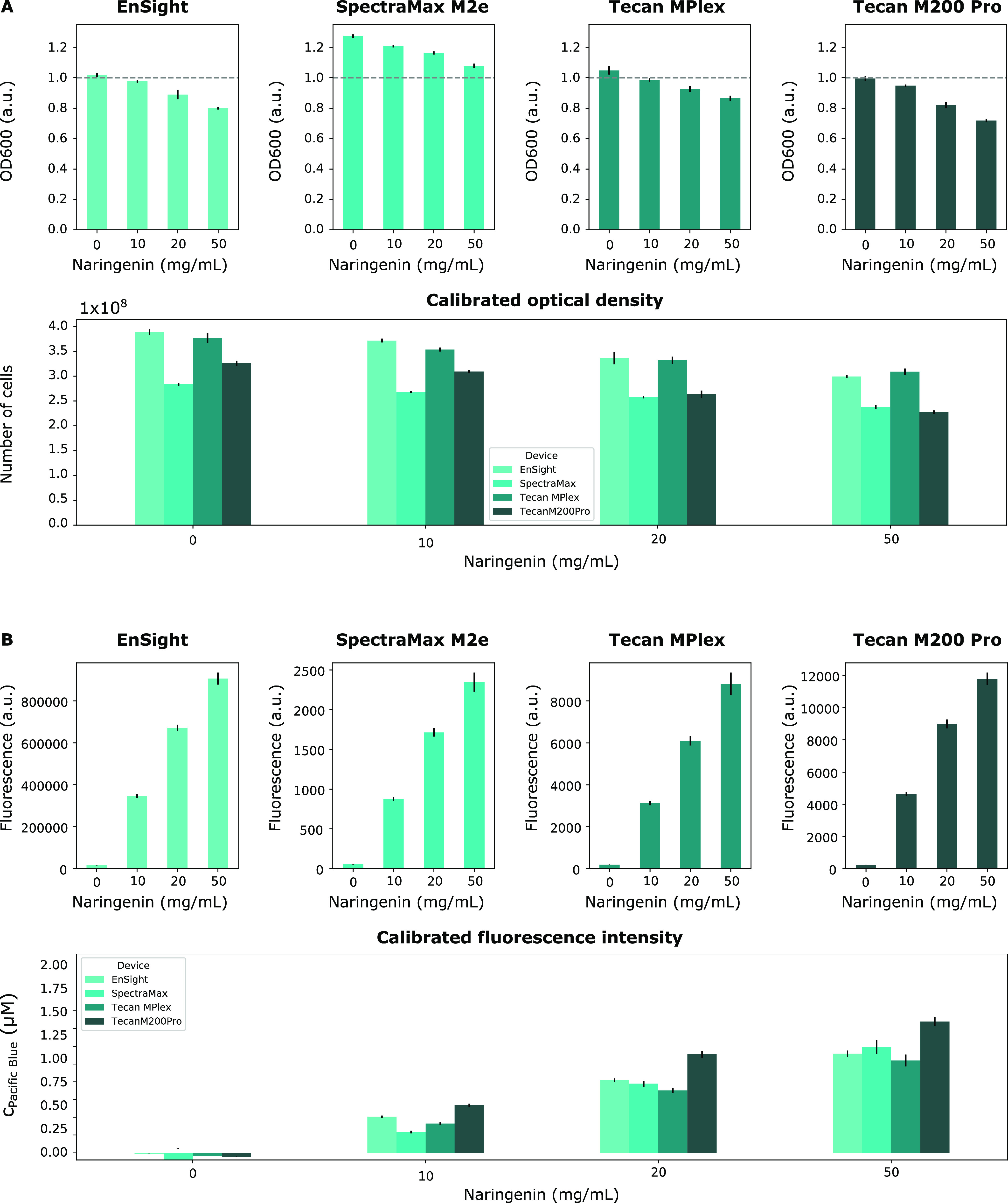
Results of the optical density (A) and fluorescence intensity (B) measurements of the mTagBFP-expressing strains induced using a range of naringenin concentrations (0, 10, 20, and 50 mg/L). Measurements were taken with the different plate readers: EnSight, SpectraMax M2e, Tecan MPlex, Tecan M200 Pro both before and after calibration with the (A) silica sphere calibration curves (Figure 4) and (B) Pacific Blue calibration curves (Figure 6). Error bars are standard errors and *n* = 3. Statistical analysis was performed using one-way ANOVA tests followed by a post hoc Tukey honestly significant difference (HSD) test if required (Table S7). OD600 = optical density measured at 600 nm, a.u. = arbitrary units, c_PacificBlue_ = concentration of Pacific Blue.

As a first step of the standardization protocol, the optical density values obtained for the different strains at different inducer concentrations were converted to exact amounts of cells using the appropriate calibration curves. Figure 8A gives an overview of the optical density values obtained for the EnSight, SpectraMax M2e, Tecan MPlex, and Tecan M200 Pro plate readers for the strain expressing mTagBFP. The results of the strains expressing mKate2, mCherry, OFP, sYFP2, and sfGFP can be found in Figures S12–S16A. From these results, it can be seen that the optical density decreases with increasing inducer concentrations. Since this decrease is not seen with the negative control, which is subjected to the same inducer concentrations, this decrease in growth can be attributed to the burden imposed on the cell due to the expression of the fluorescent reporters using the naringenin-inducible promoter. This effect is especially prominent for strains expressing sYFP2. A second element that should be noted from these results is that similar to the results obtained during the optical density measurements of the silica beads, the optical density values generated by the SpectraMax M2e plate reader are higher compared with the data returned by the other devices. Since the operational range of this device has already exceeded (OD600 > 1) during the exponential growth phase, it is no longer suited for this specific application.

After calibration, the optical density values fall within the same order of magnitude (Figure 8A). However, it must be noted that the cell counts obtained for the SpectraMax M2e and Tecan M200 Pro plate readers are approximately 1.2-fold lower than those of the EnSight and Tecan MPlex plate readers. A similar pattern can be seen for the cell counts obtained for the strains expressing the other fluorescent reporters. Since both the EnSight and Tecan MPlex plate readers were purchased within one year before the measurements were taken, the deviating results obtained with the SpectraMax M2e and Tecan M200 Pro plate readers, which are the oldest devices in this study, were attributed to decreased sensitivity due to the higher life span of the device and overall lower quality of measurements taken by older generation plate readers.

#### Standardization of Fluorescence Measurements between Different Plate Readers

Next, the fluorescence intensities measured by the selected devices were calibrated using the appropriate calibration curves to obtain a known concentration of equivalent fluorophore per well. The fluorescence expression of the strains expressing mTagBFP, measured by the different plate readers before and after calibration, can be seen in Figure 8B. The results confirm the differences in fluorescence output expressed in arbitrary units reported by the different devices. Although the differences between the devices are not insignificant, the resulting concentrations of Pacific Blue for the different inducer concentrations are within the same order of magnitude for the different devices. A similar trend can be seen for the strains expressing mKate2, mCherry, OFP, sYFP2, and sfGFP (Figures S12–S16). For the two red fluorescent proteins, mKate2 and mCherry, it is evident that Texas Red performs better as a calibration fluorophore for both fluorescent proteins since it results in more comparable equivalent fluorophore levels between the different plate readers when compared to the results obtained using ROX. Therefore, Texas Red is selected as the preferred fluorophore for the red fluorescent for the following data analysis.

#### Combining the Calibrated Cell Count and Fluorophore Concentrations

**Figure 9:**
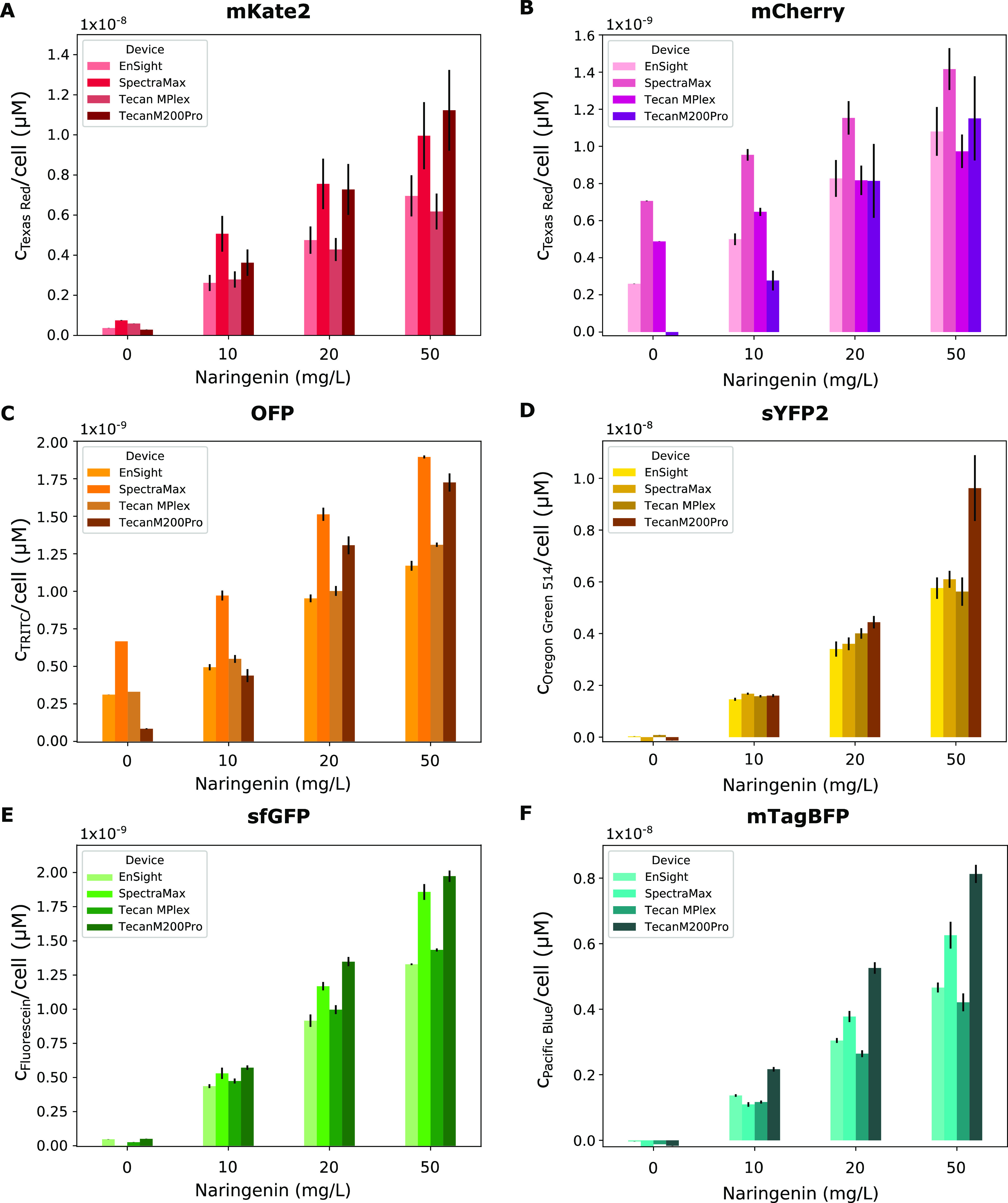
Comparison of the equivalent fluorophore concentrations per cell (μM) between the different plate readers obtained for the *E. coli* strains expressing (A) mKate2, (B) mCherry, (C) OFP, (D) sYFP2, (E) sfGFP, and (F) mTagBFP induced using different naringenin concentrations. Error bars are standard errors and *n* = 3. Statistical analysis was performed using one-way ANOVA tests followed by post hoc Tukey test if required (Tables S2–S7).

Finally, the obtained cell counts and equivalent chemical fluorophore concentrations were transformed into powerful units of equivalent fluorophore concentration per cell by dividing the equivalent fluorophore concentrations by the cell counts (Figure 9). The obtained equivalent fluorescence concentrations are in the same order of magnitude for the different fluorescent reporters for every inducer concentration. The similarity is especially apparent for the EnSight and Tecan MPlex plate readers. Deviations in the obtained equivalent fluorophore concentrations are mainly existing for the two older plate readers, namely, the SpectraMax M2e and the Tecan M200 Pro, indicating that the instrument factor has not completely been eliminated. Another remark that must be made based on the presented data is that both Texas Red and TRITC used to standardize mCherry and OFP, respectively, show a higher sensitivity compared to the other fluorophores, since a low equivalent fluorophore concentration is already obtained when no inducer is added to the strains. This could be caused by an inherent dye behavior or pipetting errors propagated through the dilution series, making the equivalent fluorophore concentrations less accurate for low fluorescence intensities.

**Figure 10:**
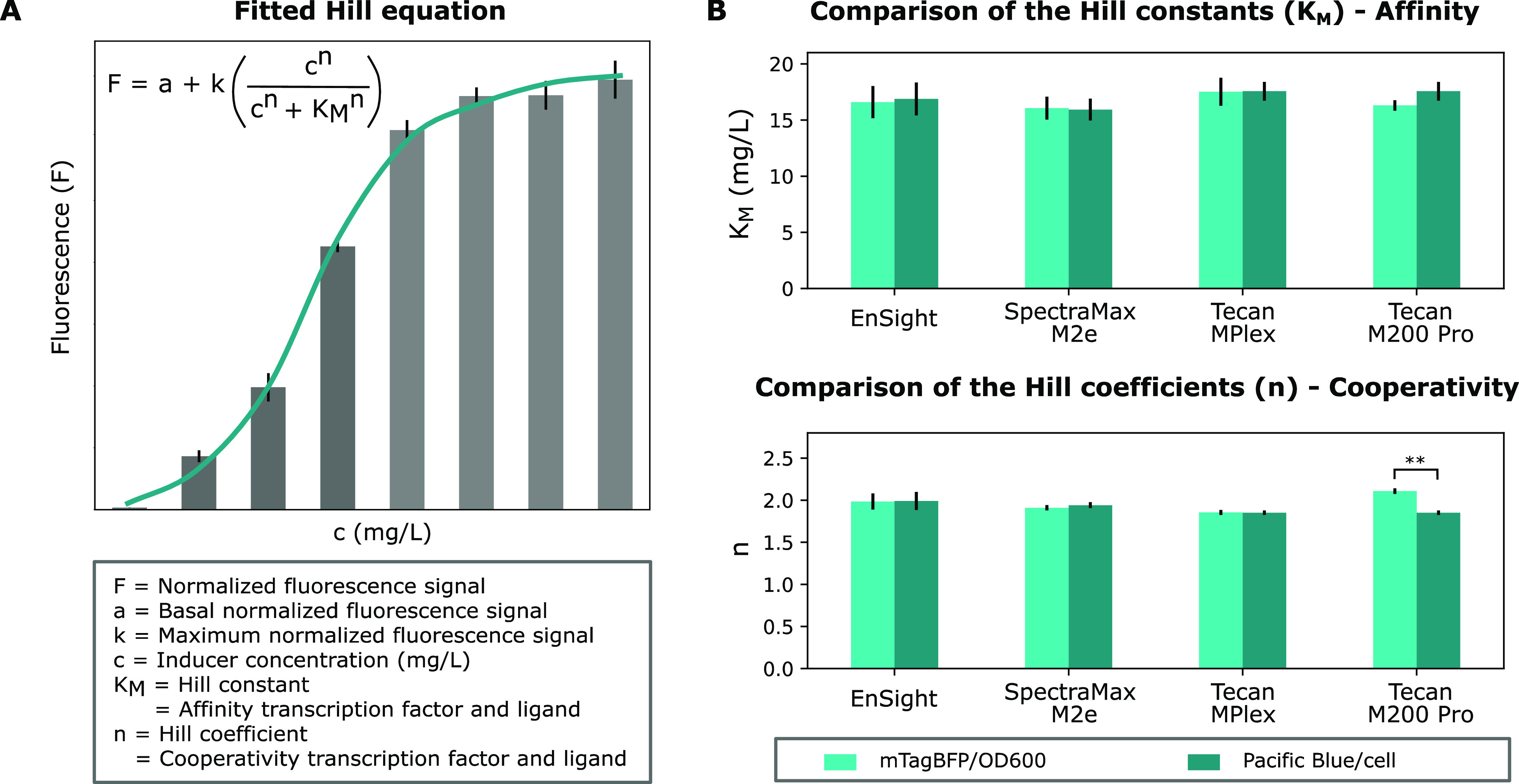
The Hill equation was used to fit a curve against the normalized fluorescence expression levels obtained before and after calibration. (A) Schematic overview of a fitted Hill equation and the corresponding parameters. (B) Comparison of the Hill constants (*K*_M_) and Hill coefficients (n) obtained after fitting the Hill equation to the data obtained for the mTagBFP-expressing *E. coli* strains. Error bars are standard errors and *n* = 3. Statistical analysis was performed using a one-way ANOVA test. ** = *p*-value <0.01.

Next, to determine whether the standardization protocol influences the intrinsic parameters associated with the behavior of the system, the Hill equation was fitted to the fluorescence levels obtained using the different devices before and after applying the calibration protocol (Figure 10A).^16^Figure 10B displays the Hill constants (*K*_M_) and the Hill coefficients (*n*) obtained after fitting the Hill equation to the strains expressing mTagBFP. An overview of the Hill parameters obtained for the other fluorescent reporters is given in Figure S17. Statistical analysis was conducted to compare the similarity of the parameters before and after implementation of the standardization protocol and between the different devices using one-way ANOVA tests. This analysis revealed that the obtained parameters do not significantly differ from each other, except for the cooperativity (n) on the data measured for mKate2, OFP, and mTagBFP using the Tecan M200 Pro and the affinity (*K*_M_) measured for mKate2 also determined using the Tecan M200 Pro. Although these differences are significant, it is argued that they are relatively small and therefore do not hamper the utility of this standardization method.

## Conclusions

During this study, a standardization protocol allowing the comparison of plate reader assays using fluorescent reporters across the visible light spectrum was developed. The protocol consists of two main calibration steps converting, first, the optical density (a.u.) into a known number of cells and, second, the fluorescence intensity (a.u.) into a known concentration of equivalent fluorophore (μM). These calibrated units were then successfully combined to express the fluorescence intensity per cell as the equivalent fluorophore concentration per cell (μM).

To optimize the first part of the calibration protocol, four different cell count methods were evaluated for their accuracy, ease of use, and affordability for all laboratories. Flow cytometry is the most accurate method; however, since this is an expensive method that is not readily available to all laboratories, it was used solely to set the reference cell count value to determine the trueness of the other methods and not suggested as the best-suited method for this calibration step. The silica spheres were selected as the preferred calibration method since they resulted in the best cell count compared to flow cytometry and additionally scored well in terms of precision. Additionally, this method scored well for ease of use since it only requires dissolution and dilution of a silica sphere solution, followed by an optical density measurement. However, one note that must be made is that the silica spheres sediment rapidly. It is important that the operator is aware of this downside since thorough mixing followed by rapid pipetting is required to obtain a high-quality calibration curve.

For the calibration of the fluorescence intensity, on the other hand, six different fluorophores were selected based on their spectral properties, price, and stability. Texas Red was chosen for mKate2 and mCherry, TRITC for OFP, Oregon Green 514 for sYFP2, sodium fluorescein for sfGFP, and Pacific Blue for mTagBFP. Each of these fluorophores was successful at converting the fluorescence intensities reported by different plate readers with 375-fold differences into equivalent fluorophore intensities that fell within the same μM order of magnitude for the different plate readers. Additionally, the same proportions between the different expression signals were maintained after calibration.

Based on the data presented for mKate2 and mCherry, one fluorophore, namely, Texas Red, can be used to calibrate multiple fluorescent proteins within the same color range. Although this could be a promising indication for the other fluorophores, further validation is required to assess whether TRITC, Oregon Green 514, fluorescein, and Pacific Blue can be used for the standardization of all experiments in which, respectively, orange, yellow, green, and blue fluorescent proteins are expressed. Additionally, an important remark that must be made is that even though the fluorophores possess the appropriate spectral properties for this application, only ROX and sodium fluorescein could be stored for 14 days without a significant change in fluorescence intensity. It is therefore important that the calibration curves are measured on the same day that the fluorophore solutions are made to ensure the correct correlation of the fluorescence intensities with the fluorophore concentration.

Overall, this method resulted in equivalent fluorophore per cell outputs within the same order of magnitude for the different plate readers tested in this study. However, some of the differences between the plate readers were significant, especially when comparing older devices to recently purchased ones. This difference might be attributed to the life span of the light sources, poor maintenance, or general improvements in the plate reader technology. These differences must be considered because, in reality, not all laboratories use brand-new plate readers. This variability is, therefore, representative of the actual results that will be obtained with this standardization protocol. To minimize the differences between devices, it is important that all laboratories should have an appropriate device maintenance and calibration schedule. An additional remark that must be made is that all plate readers calibrated in this study are monochromators, allowing for selection of the exact same excitation and emission wavelengths for all fluorescence measurements. A large amount of plate readers used contain a fixed set of filters, limiting the flexibility of wavelength selection. Moreover, since in this study, the exact same fluorophore dilutions and cell culture plates that were prepared by one single operator were measured on the four plate readers, an additional level of variability will be introduced when this protocol is validated by different operators in laboratories across the world. Therefore, to fully validate this protocol, it should be tested through a collaborative assessment experiment across laboratories.

Furthermore, the standardization protocol presented here is a promising starting point for the development of calibration protocols for different microbial hosts. This is especially interesting since several researchers in synthetic biology are exploring nonstandard hosts for a wide variety of applications.^17^ By selecting the appropriate size of the spheres, this method could be converted into a standardization protocol for a range of different interesting microbial hosts.

## Materials and Methods

### Strains and Growth Media

The cloning steps were performed using *E. coli* TOP10 cells (Invitrogen, Carlsbad). *E. coli* K12 MG1655 (ATCC 47076) was used for growth experiments. Cells were made electrocompetent using the glycerol/mannitol density gradient wash protocol.^18^ After DNA was added, the cells were electroporated at 1.8 kV, 200 Ω, and 25 μF in a prechilled electroporation cuvette (Bio-Rad) with a 1 mm gap-width. After electroporation, 900 μL of LB was added for regeneration, followed by plating on LB-agar plates containing the required antibiotic. All products were purchased from Merck (Diegem, Belgium), unless mentioned otherwise. Lysogeny Broth (LB) containing 10 g/L bactotryptone, 10 g/L NaCl, and 5 g/L yeast extract was used for cloning purposes. LB-agar plates consisted of LB supplemented with 12 g/L agar. All growth experiments were performed using defined medium containing 2 g/L NH_4_Cl, 5 g/L (NH_4_)2SO4, 3 g/L KH_2_PO_4_, 7.3 g/L K_2_HPO_4_, 8.4 g of MOPS, 0.5 g/L MgSO_4_.7H_2_O, 16.5 g/L glucose.H_2_O, 1 mL of trace element solution, and 100 μL of molybdate solution. The pH was set to 7 using KOH prior to autoclaving for 30 min at 121 °C. The trace element solution consisted of 3.6 g of FeCl_2_.4H_2_O, 5 g of CaCl_2_.2H_2_O, 1.3 g of MnCl_2_.2H_2_O, 0.38 g of CuCl_2_.2H_2_O, 0.5 g of CoCl_2_.6H_2_O, 0.94 g of ZnCl_2_, 0.0311 g of H_3_BO_4_, 0.4 g of Na_2_EDTA.2H_2_O, and 1.01 g of thiamine HCl per liter, and the molybdate solution contained 0.967 g/L Na_2_MoO_4_.2H_2_O. The MgSO_4_.7H_2_O and glucose were autoclaved separately from the other salts to avoid Maillard reaction and precipitation of the salts. The trace element and molybdate solutions were filter sterilized by using a 0.22 μm bottletop filter (Corning PTFE filter) and added just before use. If required, the growth medium was supplemented with kanamycin to a final concentration of 50 μg/mL and incubation happened at 30 °C.

### Plasmid Construction

All six different fluorescent reporters mKate2, mCherry, OFP, sYFP2, sfGFP, and mTagBFP were cloned downstream of a naringenin-inducible promoter (P_fdeA_) on a pBBR1-MCS2 vector. In addition to these vectors, a vector consisting of only the pBBR1-MCS2 backbone was created to serve as a negative control. The coding sequences of *mKate2*, *sYFP2*, and *sfGFP* were available in-house, *mCherry* was a gift from Baldwin lab (Imperial College, London, United Kingdom), and *OFP* and *mTagBFP* were isolated from the 2016 iGEM Distribution Kit. P_fdeA_ was developed in-house,^16^ and the pBBR1-MCS2 vector was a gift from the MICR research group (Vrije Universiteit Brussel, Brussels, Belgium). The coding sequences of the fluorescent reporters and P_fdeA_ were amplified using PrimeSTAR HS DNA polymerase (Takara, Westburg, Leusden, The Netherlands), and the backbone was amplified using PrimeSTAR GXL DNA polymerase (Takara). Subsequently, the polymerase chain reaction (PCR) products were purified using the innuPREP PCRpure Kit (Analytic Jena AG, Jena, Germany) and assembled using Circular Polymerase Extension Cloning (CPEC)^19^ with Q5 DNA polymerase (New England BioLabs, Ipswich, MA) in the case of the constructs containing the fluorescent reporters and Golden Gate assembly (GGA)^20^ for the negative control. The newly assembled plasmids were then introduced into *E. coli* TOP10 (Invitrogen, Carlsbad, California) and verified using colony PCR. For each plasmid, one positive colony was grown overnight, followed by plasmid purification using the QIAprep Spin Miniprep Kit (Qiagen, Venlo, The Netherlands) and Sanger sequencing to verify the DNA-sequence (Macrogen, Inc., Amsterdam, The Netherlands). Once the correct sequence was confirmed, the plasmids were introduced into *E. coli* K12 MG1655 (ATCC 47076) for growth testing. A list of all oligonucleotides (IDT, Leuven, Belgium) used during this project is given in Table S8.

### Cell Count Methods

The cell count methods were performed on cultures of *E. coli* K12 MG1655 containing pBBR1-MCS2 (negative control). Three biological replicates were grown. First, a streak from the glycerol stock was incubated for 24 h at 30 °C. Next, a single colony was inoculated at 30 °C in a test tube containing LB supplemented with kanamycin. After 24 h, 1 mL of this initial culture was inoculated in 100 mL of LB containing kanamycin in a 500 mL shake flask and incubated for 24 h at 30 °C at 200 rpm on an orbital shaker (AppliTek, Nazareth, Belgium). This culture was used as a starter culture, of which 100 μL was inoculated in 100 mL of defined medium containing kanamycin in a 500 mL shake flask. Once the cell culture reached the required OD600 of approximately 3.5 (mid-exponential growth phase), they were immediately dispensed in 50 mL tubes and stored on ice to stop cell growth. All cell count methods described below were performed in duplicate to obtain two technical replicates.

#### Hemocytometry

Prior to dilution, all samples were mixed well to ensure equal distribution of the cells and stained using methylene blue to allow the distinction between damaged and intact cells. Next, the samples were diluted to avoid overlapping of the cells in the case of a too dense sample or higher statistical errors in the case of a too diluted sample. The samples of the exponential phase cell culture were diluted 10x. These dilutions were made using 0.9% saline solution to avoid the bursting of the cells. Next, 10 μL of the diluted sample was loaded into the counting chamber (Marienfeld, Lauda-Königshofen, Germany). Coverslips suitable for hemocytometry were tested (Bright-Line Hemocytometer coverslip) but not found to be suitable due to a blurred and dark image caused by the thickness of the glass. These special coverslips are thicker than regular coverslips, avoiding inflection due to capillary forces of the sample in the hemocytometer. Therefore, using these coverslips guarantees the exact volume of the square. However, due to blurred vision caused by the thickness of the hemocytometer coverslips, these were not suitable for counting *E. coli* cells at the required magnification (100×). Hence, regular coverslips were used (18 mm × 18 mm, Marienfeld, Lauda-Königshofen, Germany). After the coverslip was placed on the counting chamber, the sample was allowed to set for 4 min. Once set, three squares with a volume of 4 nL were counted for every technical replicate. Cells touching the top and right borders of the grid were not counted, while cells touching the bottom and left edges were taken into account. The following formula was used to determine the number of cells present in the original growth cultures:
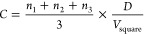
where *C* represents the concentration of cells in the original cell culture (cells/mL); *n*_1_, *n*_2_, and *n*_3_ are the number of cells counted in the three squares; *D* is the dilution factor; and *V*_square_ is the volume of one square (mL). Important to note here is that the number of damaged cells was negligible compared to the number of intact ones.

#### Cell Dry Weight Determination

To prepare for the cell dry weight measurements, 50 mL falcon tubes were dried for 48 h at 70 °C. Once fully dried, they were transferred to a desiccator and allowed to cool to room temperature. These predried falcon tubes were then weighed. Next, 10.00 g of the ice-cold cell culture was added and centrifuged for 20 min at 9000*g* and 4 °C. After discarding the supernatant, the cells were resuspended in 10 mL of physiological solution (0.9% NaCl) and centrifuged again for 20 min at 9000*g* and 4 °C. Subsequently, the pellet was dried for 48 h at 70 °C, after which they were transferred to the desiccator, cooled down, and weighed using an analytical balance. It must be noted that it is highly important to carefully discard all of the saline solution since leftover salt will increase the weight of the sample. Based on the weights gathered before and after adding the sample, the number of cells in the cell cultures can be calculated using the following formula
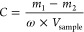
with *C* the concentration of cells in the cell culture (cells/mL), *m*_1_ and *m*_2_ the weight of the 50 mL tubes, respectively, before and after adding the sample, ω the weight of one dried *E. coli* cell in the exponential growth phase in growth medium containing glucose (2.8 × 10^–13^ g/dry cell^12^), and *V*_sample_ the volume of cell culture added to the 50 mL tube, assuming a cell culture density of 1 g/mL.

#### Silica Sphere Calibration

Silica spheres with an average diameter of 1.05 μm (Polysciences, Germany) were selected based on their similarity in size and light scattering properties when compared to *E. coli*. Knowing that there are 8.249 × 10^11^ spheres per gram, a stock solution containing 2 × 10^6^ spheres or beads per μL was made by dissolving 0.5 g of silica spheres in 206.226 mL of ddH_2_O. After adding the beads to the ddH_2_O, the solution was stirred for 2 h using a magnetic stirrer to ensure complete dissolution of the spheres. It is important to note that the sphere solution must be stirred thoroughly before use since the silica spheres tend to sediment rapidly. Next, to correlate a range of sphere concentrations to the optical density, the OD600 of a selection of dilutions spanning the linear range of the plate readers (see Table 1) was measured in octuplicate in a transparent flat-bottom 96-well plate (Greiner Bio-One, Vilvoorde, Belgium) using the four different devices included in this study (EnSight multimode plate reader (PerkinElmer, Zaventem, Belgium), SpectraMax M2e (Thermo Fisher Scientific, Erembodegem, Belgium), Tecan MPlex and Tecan M200 Pro (Tecan Benelux, Mechelen, Belgium) plate readers). The data obtained from the Tecan M200 Pro plate reader were used during the comparative study of the cell count methods. To determine the correlation curve between the measured optical density as a dependent variable and the number of cells in one well as the independent variable, linear regression was performed on the generated data using the linear_regression package in Python.

**Table 1:** Volumes Used to Prepare the Dilutions of the Silica Sphere Stock Solution^a^

**dilution**	**stock solution (μL)**	**ddH**_**2**_**O (μL)**
1	150	0
2	135	15
3	120	30
4	105	45
5	90	60
6	75	75
7	60	90
8	45	105
9	30	120
10	15	135
11	7.5	142.5
12	0	150

a These dilutions were made in octuplicate in a transparent 96-well flat-bottom plate and resulted in absorbance values distributed evenly across the linear range of optical density measurements.

#### Flow Cytometry

The samples were first prepared by diluting them 1000× in a 0.22 μm filtered PBS solution. After diluting, the samples were stained using SYBR Green I (10 000× stock, Thermo Fisher Scientific) and propidium iodide (PI, Thermo Fisher Scientific) to a final concentration of 10 μL/mL, and incubated for 20 min at 37 °C to allow the stains to set. The measurements were performed using the Attune NxT Flow Cytometer (Thermo Fisher Scientific), equipped with violet (405 nm, 50 mW) and blue (488 nm, 50 mW) laser. Events were thresholded on the BL1-H channel (530/30 bandpass filter), where the SYBR Green I fluorescence was measured. Emission of PI was measured in the BL3 fluorescence channel (695/40 bandpass filter). The samples were run at a flow rate of 100 μL/min and attention was paid to acquisition-level data quality by monitoring the fluorescence over time as well as the occurrence of nonsinglet cells. All data were extracted from the proprietary Attune NxT Flow Cytometer software (Thermo Fisher Scientific).

### Fluorophores

#### Development of the Standard Solutions

A stock solution was made for each of the selected fluorophores, sulforhodamine 101 chloride (Texas Red), Carboxy-X-Rhodamine (ROX), tetramethylrhodamine (TRITC), Oregon Green 514 (Thermo Fisher Scientific), sodium fluorescein, and Pacific Blue (Gentaur Europe, Kampenhout, Belgium) using either 10 mM PBS or DMSO as solvent. The PBS solution containing 4 g/L NaCl, 100 mg/L KCl, 0.72 g/L Na_2_HPO_4_, and 122.5 mg/L KH_2_PO_4_ was set at pH 7.4 and was the preferred solvent since it is an aqueous solution. However, in the case of low solubility or decreased stability when dissolved in PBS, DMSO was used to improve both the solubility and the stability of the fluorophores. The fluorophores were weighed using an analytical scale (Sartorius CP225D, Sartorius AG, Götingen, Germany) with an accuracy of 0.01 mg. Using a volumetric flask, the desired volume of the solvent was accurately added. An overview of the final concentrations of the stock solutions and the solvent that was used is given in Table S1. All fluorophore solutions were stored in the dark at −20 °C. To characterize the chemical fluorophores dissolved in their specific solvent, a spectrum scan was taken between 300 and 900 nm using quartz cuvettes (Hellma absorption cuvette) using a UV-1600PC spectrophotometer (VWR, Leuven, Belgium). The molar attenuation coefficient (ε) was calculated using the Beer–Lambert law:^21^
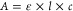
where *A* represents the attenuation of light at the wavelength at which the absorption of photon is the highest (λ_max_), ε equals the molar attenuation coefficient (cm^–1^ × M^–1^), *l* is the path length (cm), and *c* is the concentration of the fluorophore solution (M).

#### Development of the Standard Curves

Starting from these fluorophore stock solutions, standard curves were made based on the protocol established by the iGEM InterLab studies.^7,8^ The stock solutions of Texas Red, TRITC, Oregon Green 514, and sodium fluorescein were diluted to 10x reference solutions with a concentration of 100 μM, and ROX and Pacific Blue were diluted to a concentration of 500 μM using their respective solvents. These reference solutions were diluted using PBS to obtain 1x working solutions that are used as the starting point to make the dilution series in a black flat-bottom 96-well plate (Greiner Bio-One) prefilled with 100 μL PBS (Figure S18). After measuring the dilution series, the output was corrected for the background fluorescence of PBS. After this correction was performed, the correlation between the measured fluorescence (a.u.) to a known concentration of fluorophore was determined by means of linear regression using the linear_regression package in Python. The linearity of the curves was evaluated using residual analysis for devices without gain settings, namely, the EnSight (PerkinElmer) and the SpectraMax M2e (Thermo Fisher Scientific). Values exceeding the linear range of the plate readers indicated detector saturation and were discarded from the data set.

To assess the stability of the fluorophores, a new dilution series was made 14 days after the stock solution was made, measured using the EnSight plate reader (PerkinElmer), and compared to the intensities measured at day 1. The fluorescence measured at day 1 was scaled to 100%, allowing a straightforward comparison of the stability of the selected fluorophores.

### Plate Reader Assays

The fluorescence assay was performed by inoculating a preculture plate containing three different colonies of each strain (biological replicates) in 150 μL of defined medium supplemented with kanamycin in a sterile transparent 96-well flat-bottom plate (Greiner Bio-One). This preculture plate was incubated for 24 h at 30 °C while being shaken at 800 rpm (Compact Digital Microplate Shaker, Thermo Fisher Scientific). Before diluting the cell cultures for the final assay, the appropriate amount of naringenin was added to each well (0, 10, 20, or 50 mg/L). A solution containing 1 mg/mL naringenin dissolved in ethanol was used as a stock solution. After the inducer was added, the plate was left at room temperature for 1 h to let the ethanol evaporate to avoid ethanol-induced toxicity. Once evaporated, the precultures were diluted to a final inoculation percentage of 1% in a sterile black 96-well flat-bottom plate (Greiner Bio-One) and incubated for 6 h at 30 °C while being shaken at 800 rpm (Compact Digital Microplate Shaker) to obtain cell cultures in the exponential growth phase.

Four different plate readers, namely, the EnSight (PerkinElmer), SpectraMax M2e (Thermo Fisher Scientific), Tecan MPlex, and Tecan M200 Pro (Tecan Benelux), were used to perform optical density and fluorescence measurements. The optical density was measured at 600 nm and an overview of the excitation and emission wavelengths, and the gain settings used to measure the fluorescence emitted by the different fluorescent proteins and their corresponding fluorophores are given in Table 2. The SpectraMax M2e plate reader has an excitation and emission bandwidth of 9 nm; Tecan MPlex and M200 Pro both have an excitation bandwidth of 9 nm and an emission bandwidth of 20 nm. The number of flashes was set to 25, and the settle time was set to 150 ms for all devices.

**Table 2:** Settings Used to Measure the Fluorescence Emitted by the Fluorescent Reporters and Their Corresponding Fluorophores^a^

**fluorescent reporter**	**excitation wavelength (nm)**	**emission wavelength (nm)**	**gain**
mKate2	588	633	EnSight: n.a.
			Spectramax M2e: n.a.
			Tecan MPlex: 110
			Tecan M200 Pro: 90
mCherry	580	610	EnSight: n.a.
			Spectramax M2e: n.a.
			Tecan MPlex: 110
			Tecan M200 Pro: 110
OFP	543	573	EnSight: n.a.
			Spectramax M2e: n.a.
			Tecan MPlex: 110
			Tecan M200 Pro: 80
sYFP2	515	545	EnSight: n.a.
			Spectramax M2e: n.a.
			Tecan MPlex: 110
			Tecan M200 Pro: 70
sfGFP	480	520	EnSight: n.a.
			Spectramax M2e: n.a.
			Tecan MPlex: 110
			Tecan M200 Pro: 70
mTagBFP	399	456	EnSight: n.a.
			Spectramax M2e: n.a.
			Tecan MPlex: 110
			Tecan M200 Pro: 70

a There is no gain setting for both the EnSight and SpectraMax M2e plate readers since they normalize the output using an internal reference. n.a. = not applicable.

The optical density of the cell cultures was corrected for the background optical density of the growth medium. Similarly, the fluorescence measured for the strains expressing the different fluorescent reporters was corrected for the autofluorescence of *E. coli* using the negative control. Next, both the corrected optical density (a.u.) and fluorescence (a.u.) values were converted into a known number of cells and fluorophore concentrations using the appropriate calibration curves. The final concentration of equivalent fluorophore per cell was calculated by dividing the determined fluorophore concentration by the number of cells in the same well.

The response curve of the naringenin-inducible promoter was fitted using the Hill equation
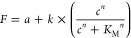
where *F* is the normalized measured fluorescence intensity; *a* is the basal normalized fluorescence signal; *k* is the maximum normalized fluorescence; *c* is the inducer concentration (mg/L); *K*_M_ is the Hill constant representing the affinity between the transcription factor, FdeR, and the ligand, naringenin; and *n* is the Hill coefficient, representing the cooperativity between the transcription factor and the ligand. Significant differences between groups were determined using one-way ANOVA tests performed using the statsmodels Python module. If required, a Tukey honestly significant difference (HSD) test was performed to assess which strains within one group significantly differ from each other.

## Supporting Information Available

Absorbance spectra of the selected chemical fluorophores (Figure S1); stability assay of the chemical fluorophores (Figure S2); calibration curves of the different chemical fluorophores measured at the selected plate readers (Figures S3–S11); raw and calibrated optical density and fluorescence results obtained for strains expressing different fluorescent reporters measured at the selected plate readers (Figures S12–S16); comparison of the Hill constants (*K*_M_) and Hill coefficients (n) obtained before and after calibration on the different plate readers (Figure S17); overview of the characteristics of the selected chemical fluorophores (Table S1); statistical analysis of the comparison of the calibrated results obtained for the different plate readers (Tables S2–S7); oligonucleotides used during strain development (Table S8); and wet lab protocol for the standardization of fluorescence plate reader assays (PDF)

## Abbreviations

au: arbitrary units
CDW: cell dry weight
*E. coli*: *Escherichia coli*
OD600: optical density measured at 600 nm
Texas Red: sulforhodamine 101 acid chloride
ROX: carboxy-X-rhodamine
TRITC: tetramethylrhodamine
