# Supplementary material for: Standardization of Fluorescent Reporter Assays in Synthetic Biology across the Visible Light Spectrum
Source: ACS Synth Biol. 2023 Nov 20;12(12):3591–607. doi: 10.1021/acssynbio.3c00386 (PMC10729763; doi:10.1021/acssynbio.3c00386)
Supplement: Supplementary file 1 — sb3c00386_si_001.pdf [file sb3c00386_si_001.pdf]

Supporting information

Standardization of fluorescence reporter assays in synthetic biology across the visible light spectrum

Lien De Wannemaeker[1], Friederike Mey[1], Indra Bervoets[2], Michiel Ver Cruysse[1], Geoff Baldwin[3] and Marjan De Mey[1]

[1] Centre for Synthetic Biology, Ghent University, Coupure links 653, 9000 Ghent, Belgium
[2] Vrije Universiteit Brussel, Pleinlaan 2, 1050 Brussels, Belgium
[3] Imperial College London, Sir Alexander Fleming Building, South Kensington, London SW7 2AZ, United Kingdom

Corresponding author: Marjan De Mey, Centre for Synthetic Biology, Ghent University, Coupure links 653, 9000 Ghent, Belgium; marjan.demey@ugent.be; +32 9 264 60 28

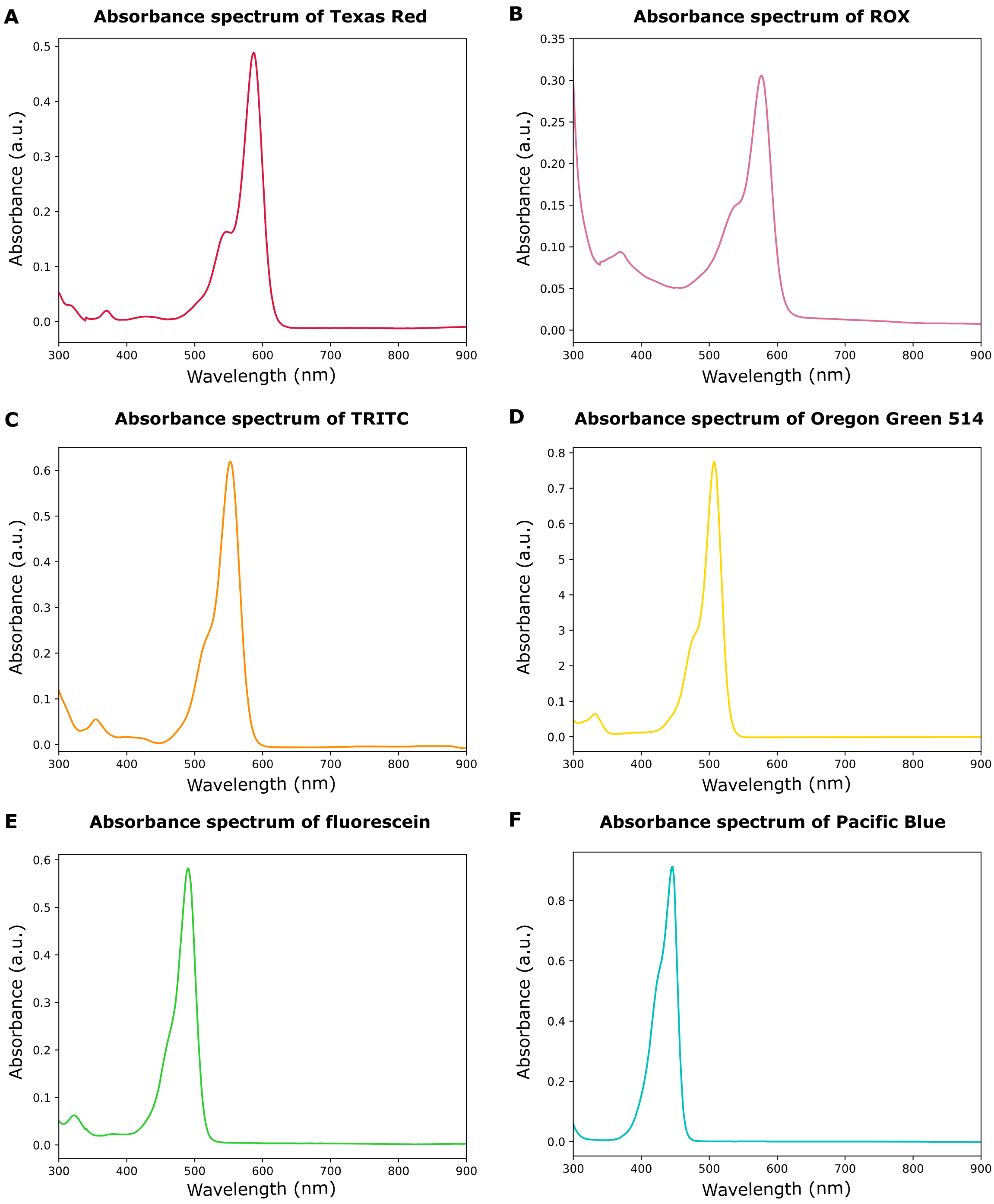

Figure S1: Absorbance spectra of the selected chemical fluorophores: (A) Texas Red (sulforhodamine 101 chloride), (B) ROX (carboxy-X-rhodamine), (C) TRITC (tetramethylrhodamine), (D) Oregon Green 514, (E) fluorescein, (F) Pacific Blue. n = 3, a.u. = arbitrary units.

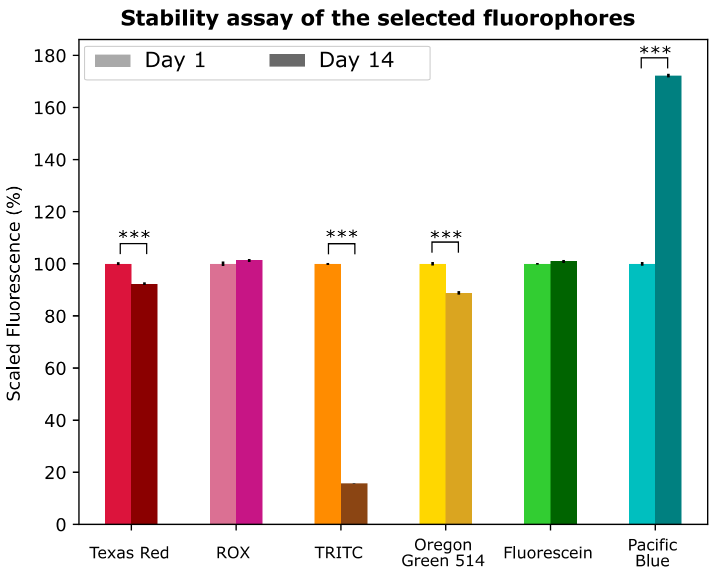

Figure S2: Scaled fluorescence intensities obtained for the different fluorophore solutions on day 1 and day 14. All measurements were preformed using the EnSight plate reader. *** = p-value < 0.001 obtained by performing a one-way ANOVA test comparing the fluorescence intensities obtained on day 1 and day 14. The values on the y-axis display the obtained fluorescence levels when the measured fluorescence at t = 0 is scaled to be 100%. Error bars are standard errors and n = 4.

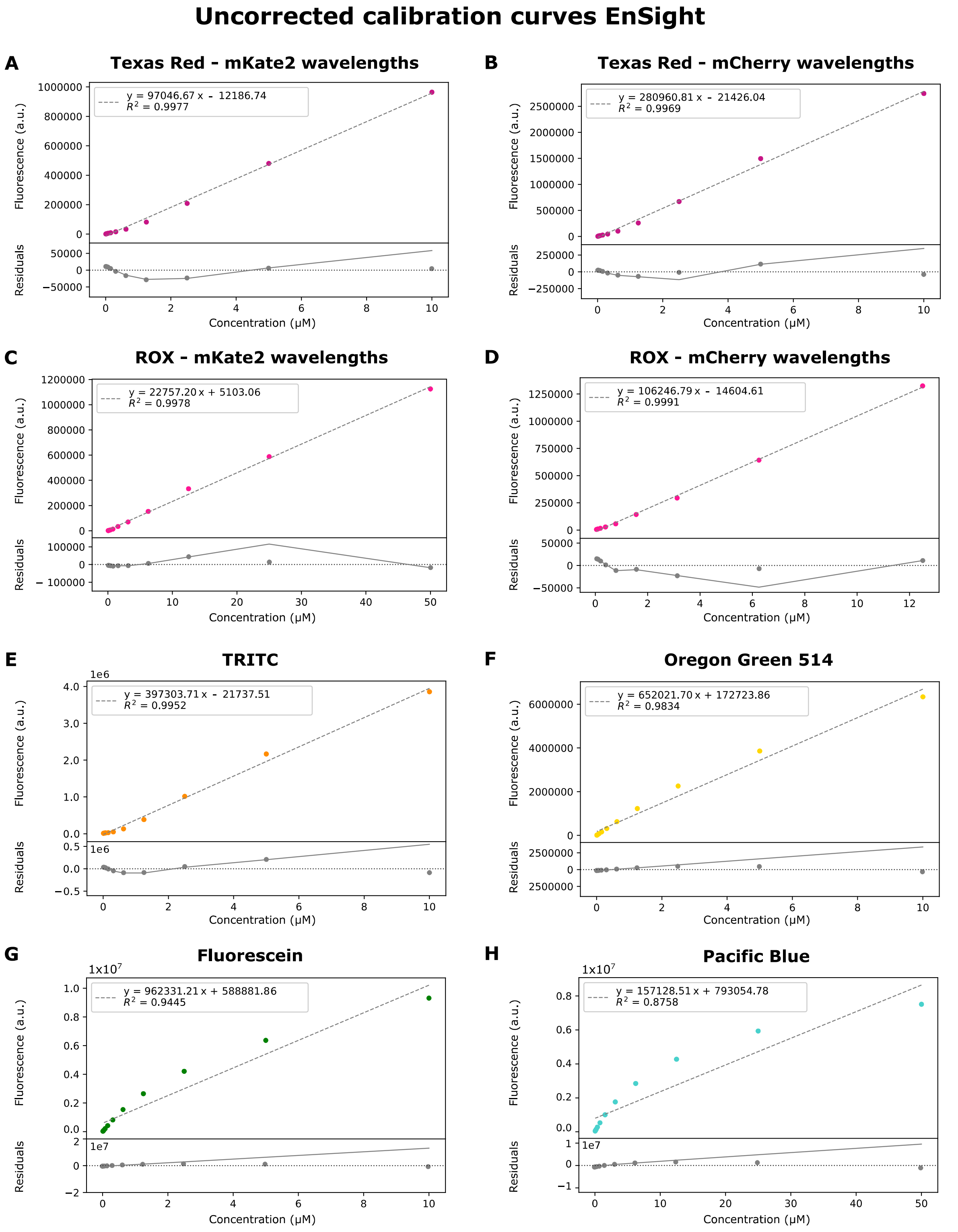

Figure S3: Calibration curves and residuals of the different fluorophore dilutions measured at the EnSight plate reader. (A) Texas Red (sulforhodamine 101 chloride) measured at 588 and 633 nm, (B) Texas Red measured at 580 and 620 nm, (C) ROX (carboxy-X-rhodamine) measured at 588 and 633 nm, (D) ROX measured at 580 and 620 nm, (E) TRITC (tetramethylrhodamine), (F) Oregon Green 514, (G) fluorescein, (H) Pacific Blue. Error bars are standard errors and n = 8. a.u. = arbitrary units.

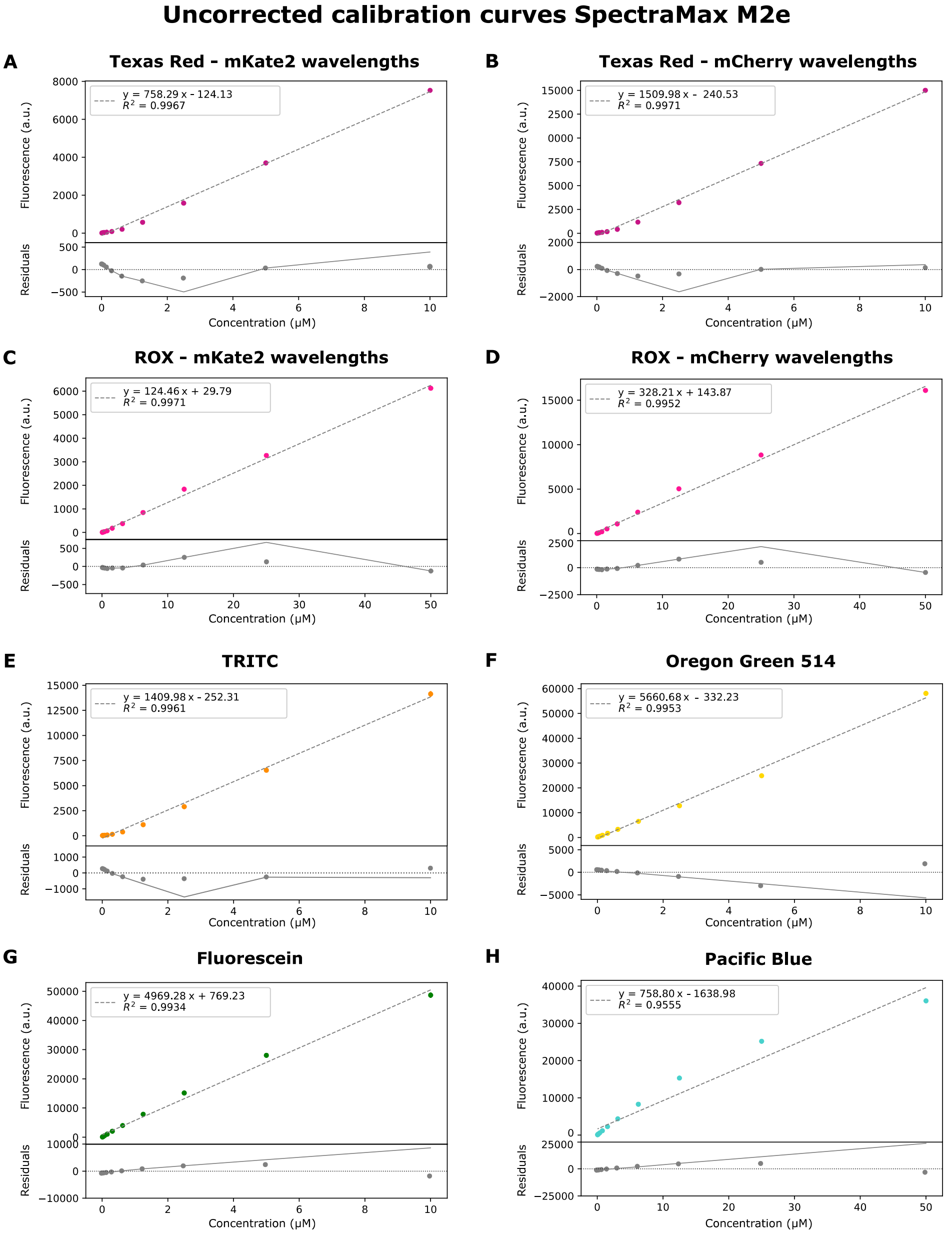

Figure S4: Calibration curves and residuals of the different fluorophore dilutions measured at the SpectraMax M2e plate reader. (A) Texas Red (sulforhodamine 101 chloride) measured at 588 and 633 nm, (B) Texas Red measured at 580 and 620 nm, (C) ROX (carboxy-X-rhodamine) measured at 588 and 633 nm, (D) ROX measured at 580 and 620 nm, (E) TRITC (tetramethylrhodamine), (F) Oregon Green 514, (G) fluorescein, (H) Pacific Blue. Error bars are standard errors and n = 8. a.u. = arbitrary units.

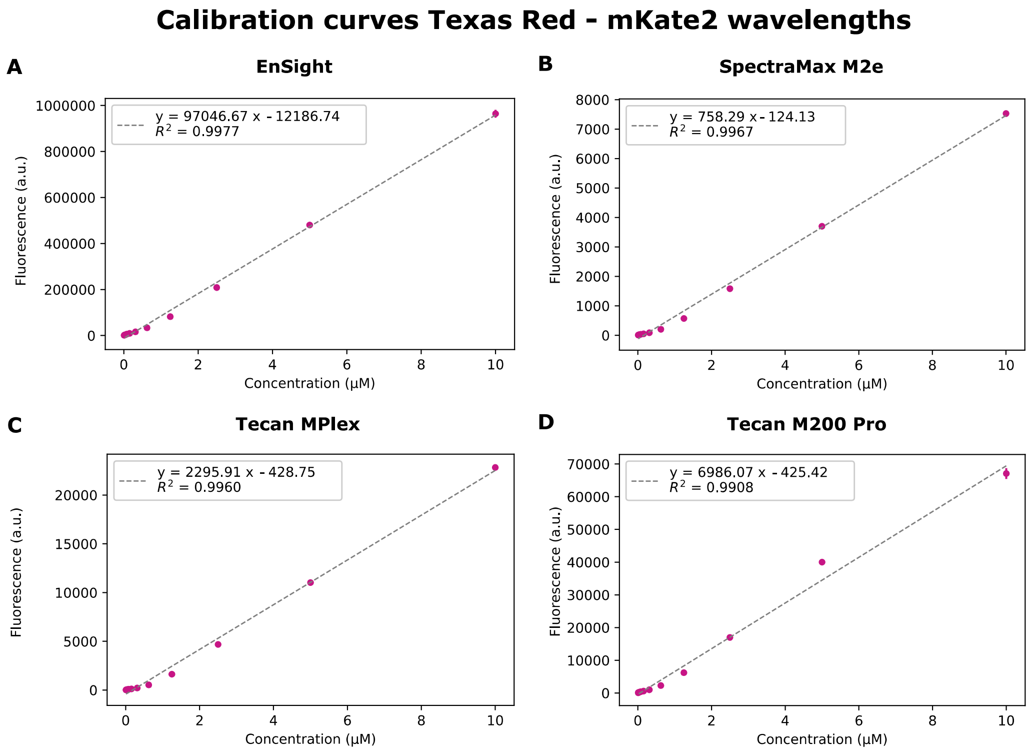

Figure S5: Calibration curves of Texas Red (sulforhodamine 101 chloride) determined for four different plate readers: (A) EnSight, (B) SpectraMax M2e, (C) Tecan MPlex, (D) Tecan M200 Pro. The measurements were taken at the optimal excitation and emission wavelengths for the red fluorescent protein mKate2 (588 and 633 nm respectively). Error bars are standard errors and n = 8. a.u. = arbitrary units.

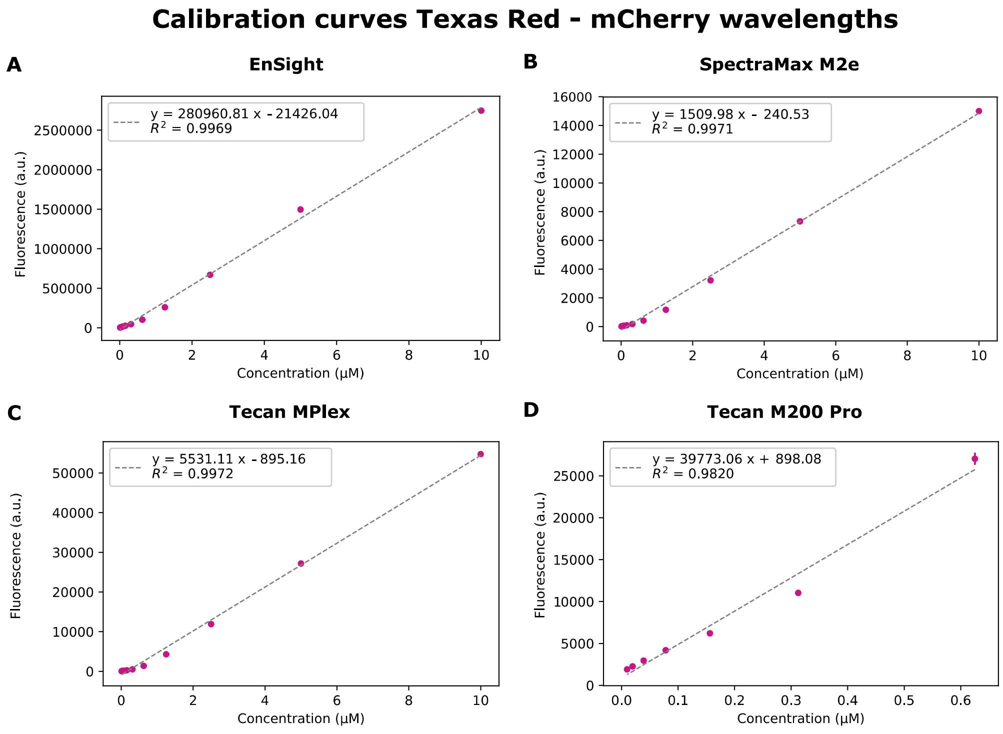

Figure S6: Calibration curves of Texas Red (sulforhodamine 101 chloride) determined for four different plate readers: (A) EnSight, (B) SpectraMax M2e, (C) Tecan MPlex, (D) Tecan M200 Pro. The measurements were taken at the optimal excitation and emission wavelengths for the red fluorescent protein mCherry (580 and 610 nm respectively). Error bars are standard errors and n = 8. a.u. = arbitrary units.

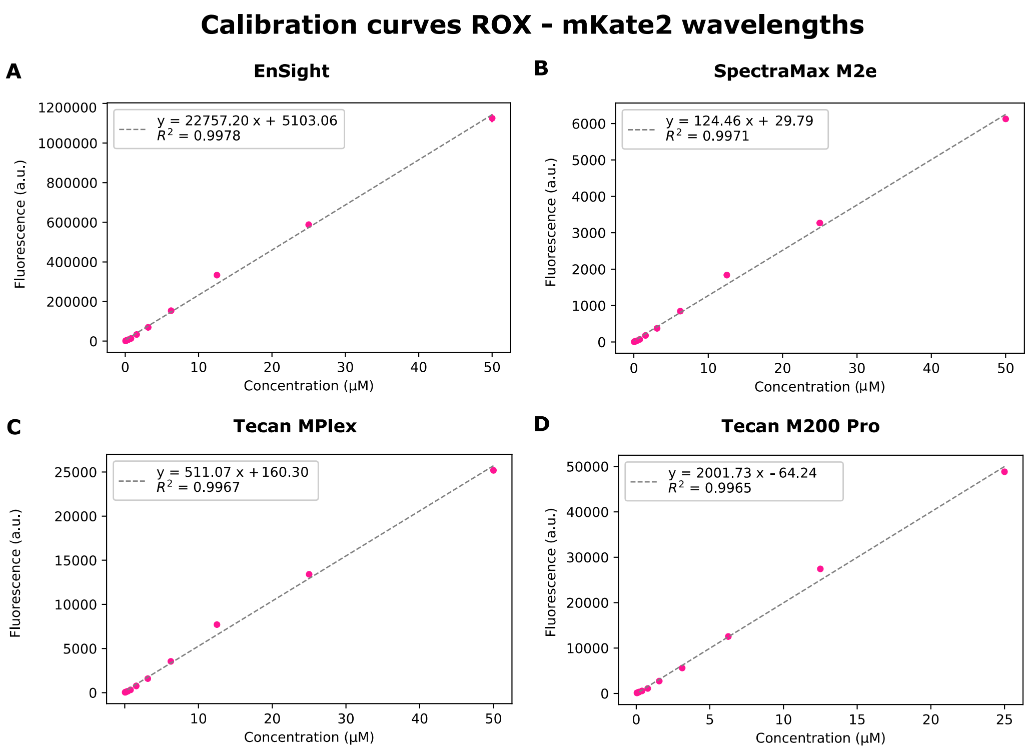

Figure S7: Calibration curves of ROX (carboxy-X-rhodamine) determined for four different plate readers: (A) EnSight, (B) SpectraMax M2e, (C) Tecan MPlex, (D) Tecan M200 Pro. The measurements were taken at the optimal excitation and emission wavelengths for the red fluorescent protein mKate2 (588 and 633 nm respectively). Error bars are standard errors and n = 8. a.u. = arbitrary units.

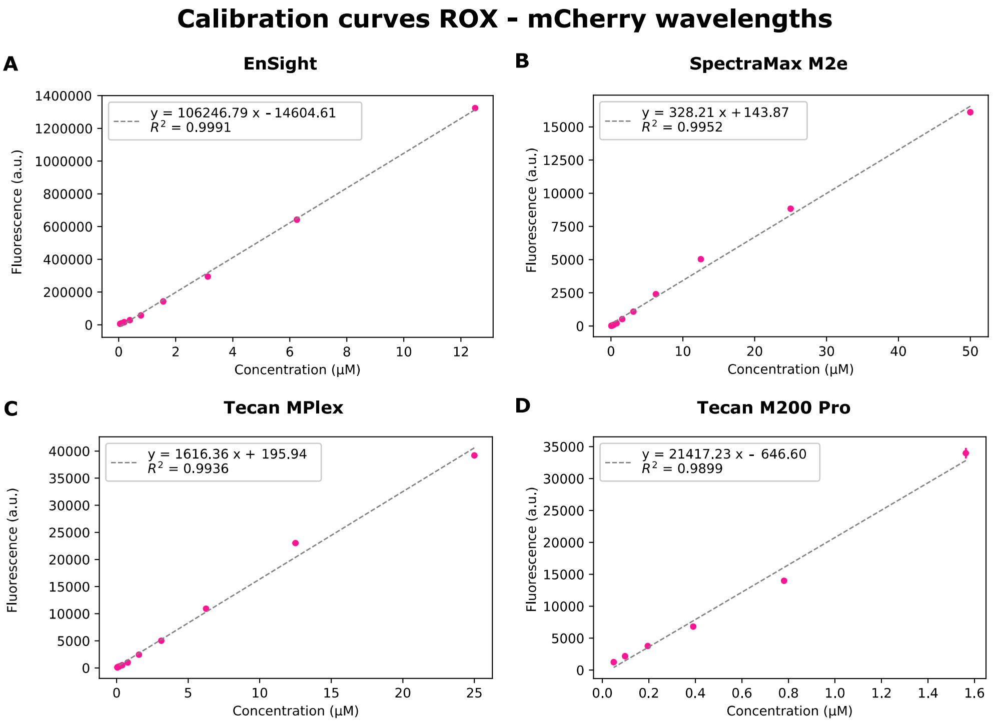

Figure S8: Calibration curves of ROX (carboxy-X-rhodamine) determined for four different plate readers: (A) EnSight, (B) SpectraMax M2e, (C) Tecan MPlex, (D) Tecan M200 Pro. The measurements were taken at the optimal excitation and emission wavelengths for the red fluorescent protein mCherry (580 and 610 nm respectively). Error bars are standard errors and n = 8. a.u. = arbitrary units.

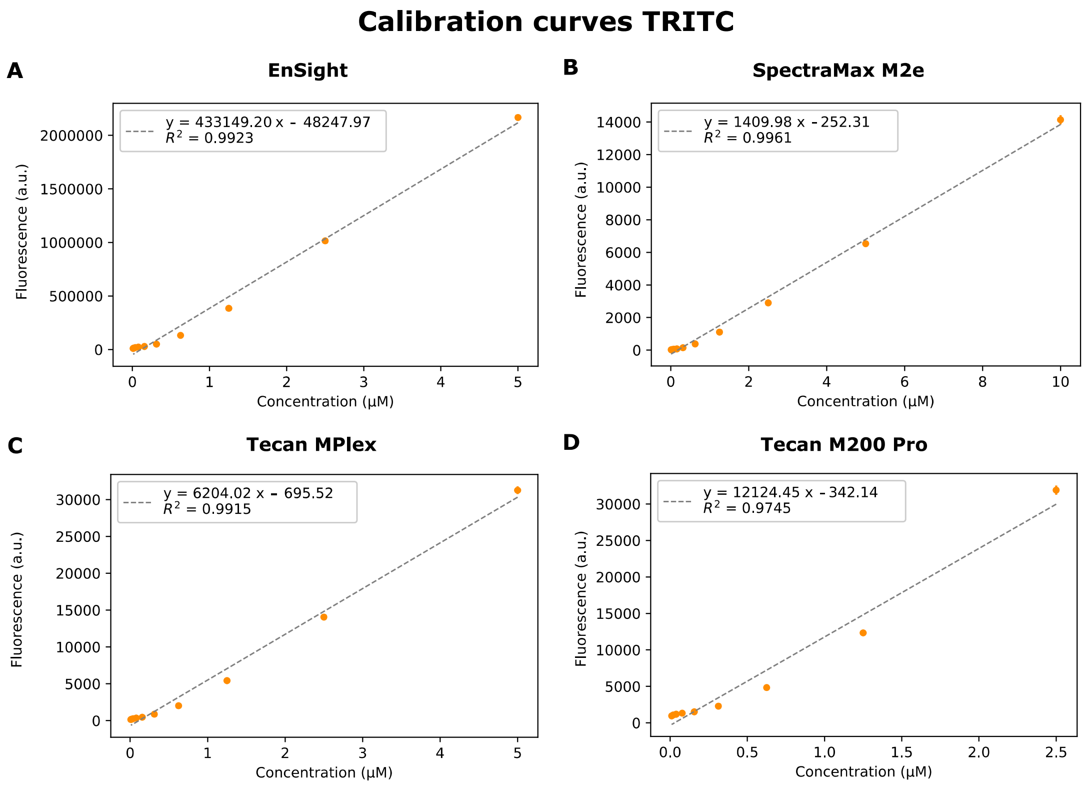

Figure S9: Calibration curves of TRITC (tetramethylrhodamine) determined for four different plate readers: (A) EnSight, (B) SpectraMax M2e, (C) Tecan MPlex, (D) Tecan M200 Pro. The measurements were taken at the optimal excitation and emission wavelengths for the orange fluorescent protein OFP (543 and 573 nm respectively). Error bars are standard errors and n = 8. a.u. = arbitrary units.

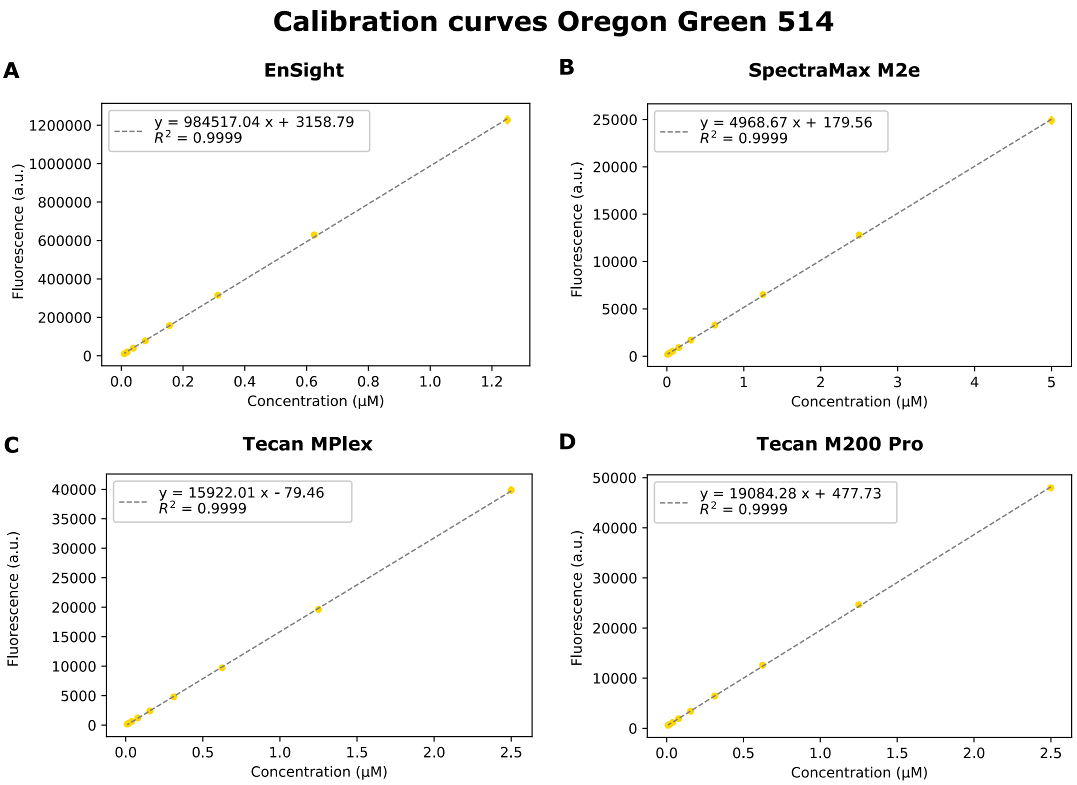

Figure S10: Calibration curves of Oregon Green 514 determined for four different plate readers: (A) EnSight, (B) SpectraMax M2e, (C) Tecan MPlex, (D) Tecan M200 Pro. The measurements were taken at the optimal excitation and emission wavelengths for the yellow fluorescent protein sYFP2 (515 and 545 nm respectively). Error bars are standard errors and n = 8. a.u. = arbitrary units.

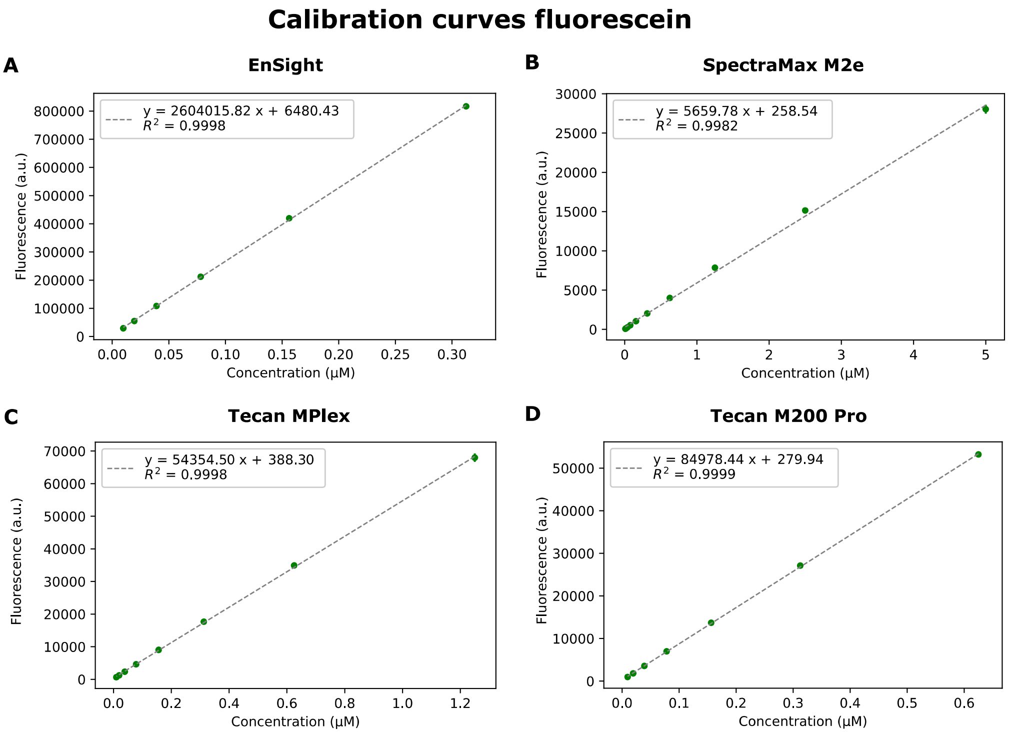

Figure S11: Calibration curves of fluorescein determined for four different plate readers: (A) EnSight, (B) SpectraMax M2e, (C) Tecan MPlex, (D) Tecan M200 Pro. The measurements were taken at the optimal excitation and emission wavelengths for the green fluorescent protein sfGFP (480 and 520 nm respectively). Error bars are standard errors and n = 8. a.u. = arbitrary units.

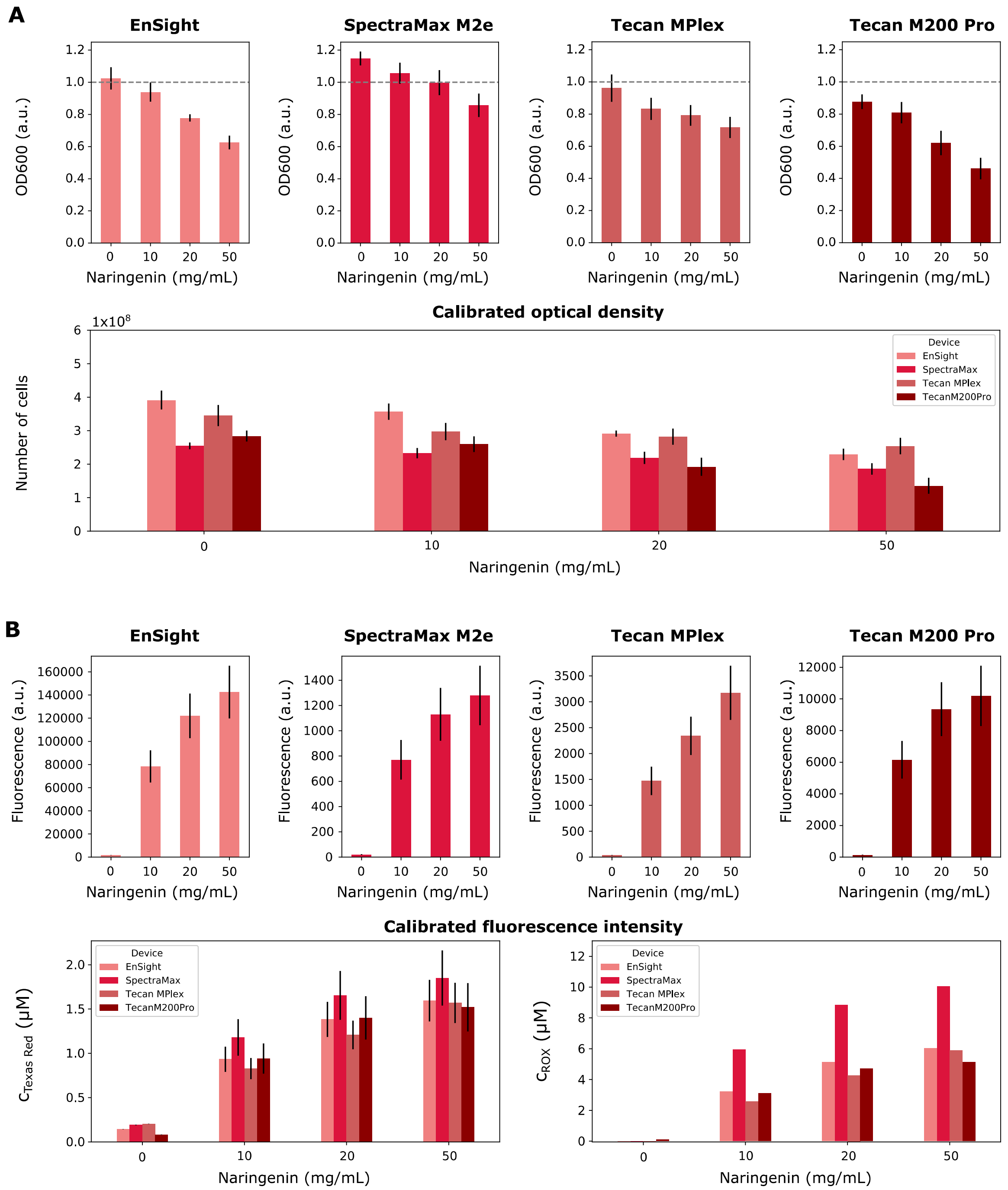

Figure S12: Results of the optical density (A) and fluorescence intensity (B) measurements of the *Escherichia coli* strains expressing the red fluorescent protein mKate2 induced by different naringenin concentrations (0, 10, 20 and 50 mg/L) measured using the different plate readers: EnSight, SpectraMax M2e, Tecan MPlex, Tecan M200 Pro both before and after calibration with the (A) calibration curves for the silica spheres (Figure 4) and (B) calibration curves determined for Texas Red (Figure S5), and ROX (Figure S7). Error bars are standard errors and n = 3. Statistical analysis was performed using one-way ANOVA tests followed by post-hoc Tukey test if required (Table S2). OD600 = optical density measured at 600 nm, a.u. = arbitrary units, $c_{Texas\ Red}$ = concentration of Texas Red, c_{ROX} = concentration of ROX.

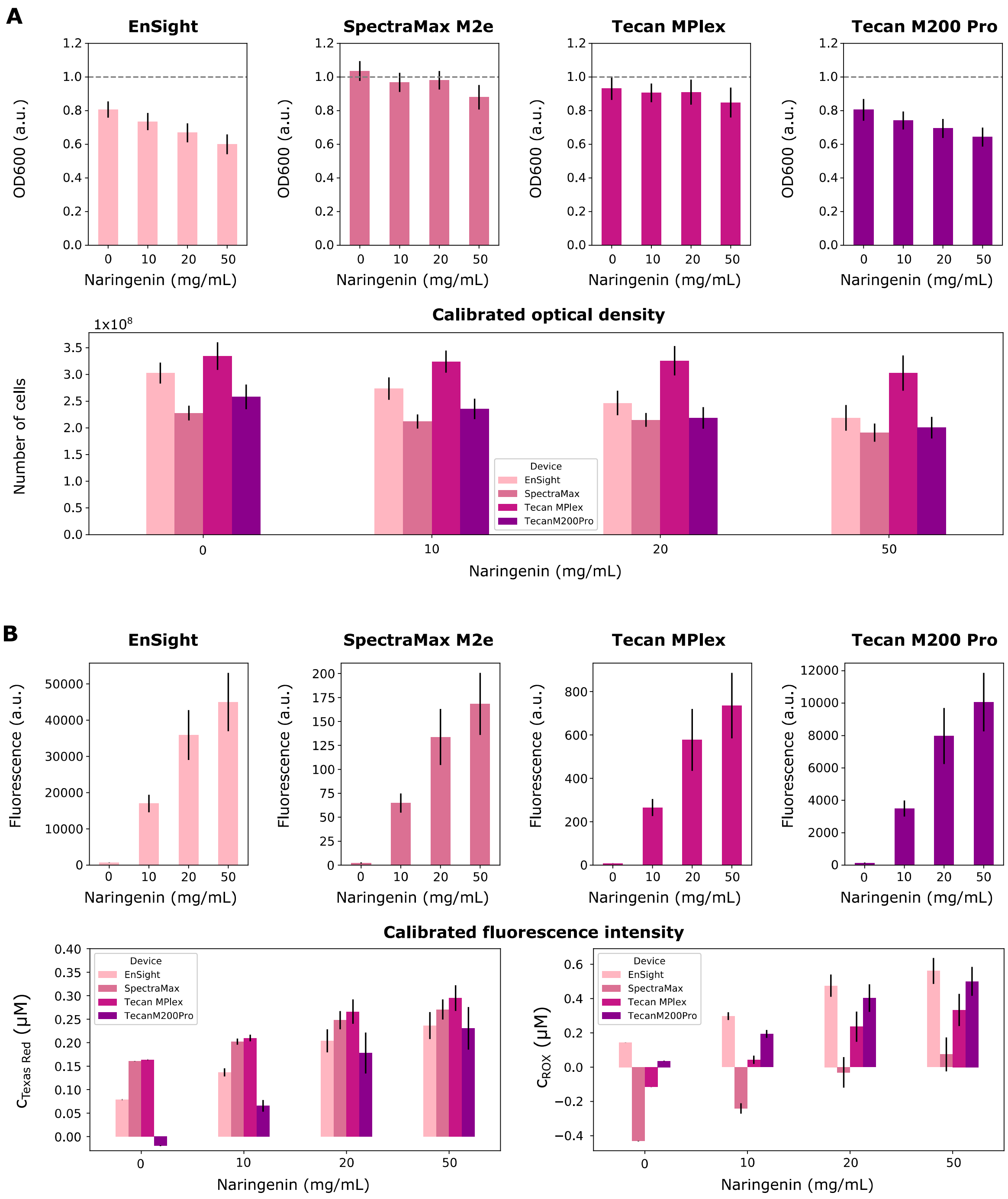

Figure S13: Results of the optical density (A) and fluorescence intensity (B) measurements of the *Escherichia coli* strains expressing the red fluorescent protein mCherry induced by different naringenin concentrations (0, 10, 20 and 50 mg/L) measured using the different plate readers: EnSight, SpectraMax M2e, Tecan MPlex, Tecan M200 Pro both before and after calibration with the (A) calibration curves for the silica spheres (Figure 4) and (B) calibration curves determined for Texas Red (Figure S6), and ROX (Figure S8). Error bars are standard errors and n = 3. Statistical analysis was performed using one-way ANOVA tests followed by post-hoc Tukey test if required (Table S3). OD600 = optical density measured at 600 nm, a.u. = arbitrary units, $c_{\text{Texas Red}}$ = concentration of Texas Red, c_{ROX} = concentration of ROX.

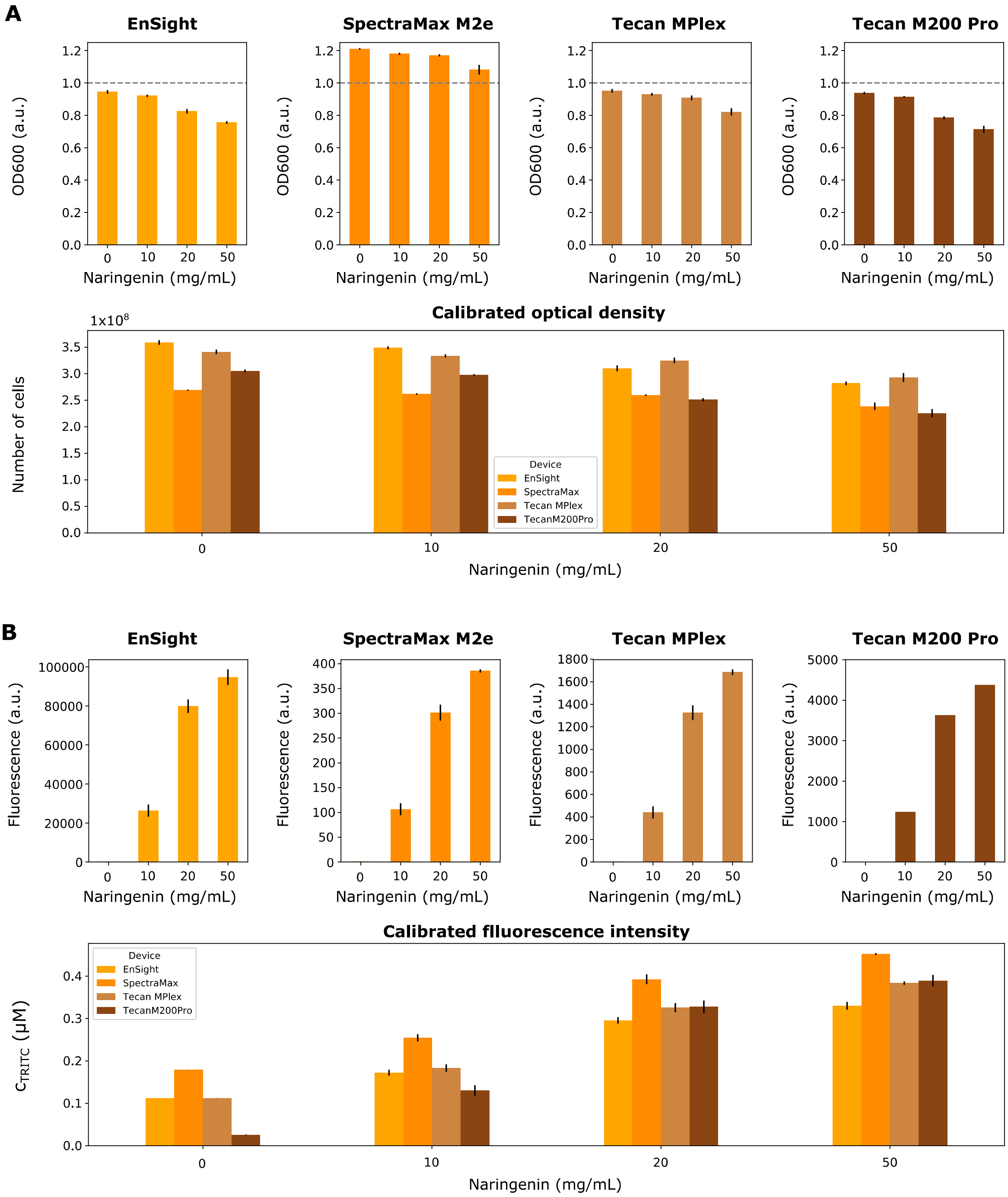

Figure S14: Results of the optical density (A) and fluorescence intensity (B) measurements of the *Escherichia coli* strains expressing the orange fluorescent protein OFP induced by different naringenin concentrations (0, 10, 20 and 50 mg/L) measured using the different plate readers: EnSight, SpectraMax M2e, Tecan MPlex, Tecan M200 Pro both before and after calibration with the (A) calibration curves for the silica spheres (Figure 4) and (B) calibration curves determined for TRITC (Figure S9). Error bars are standard errors and n = 3. Statistical analysis was performed using one-way ANOVA tests followed by post-hoc Tukey test if required (Table S4). OD600 = optical density measured at 600 nm, a.u. = arbitrary units, c_{TRITC} = concentration of TRITC.

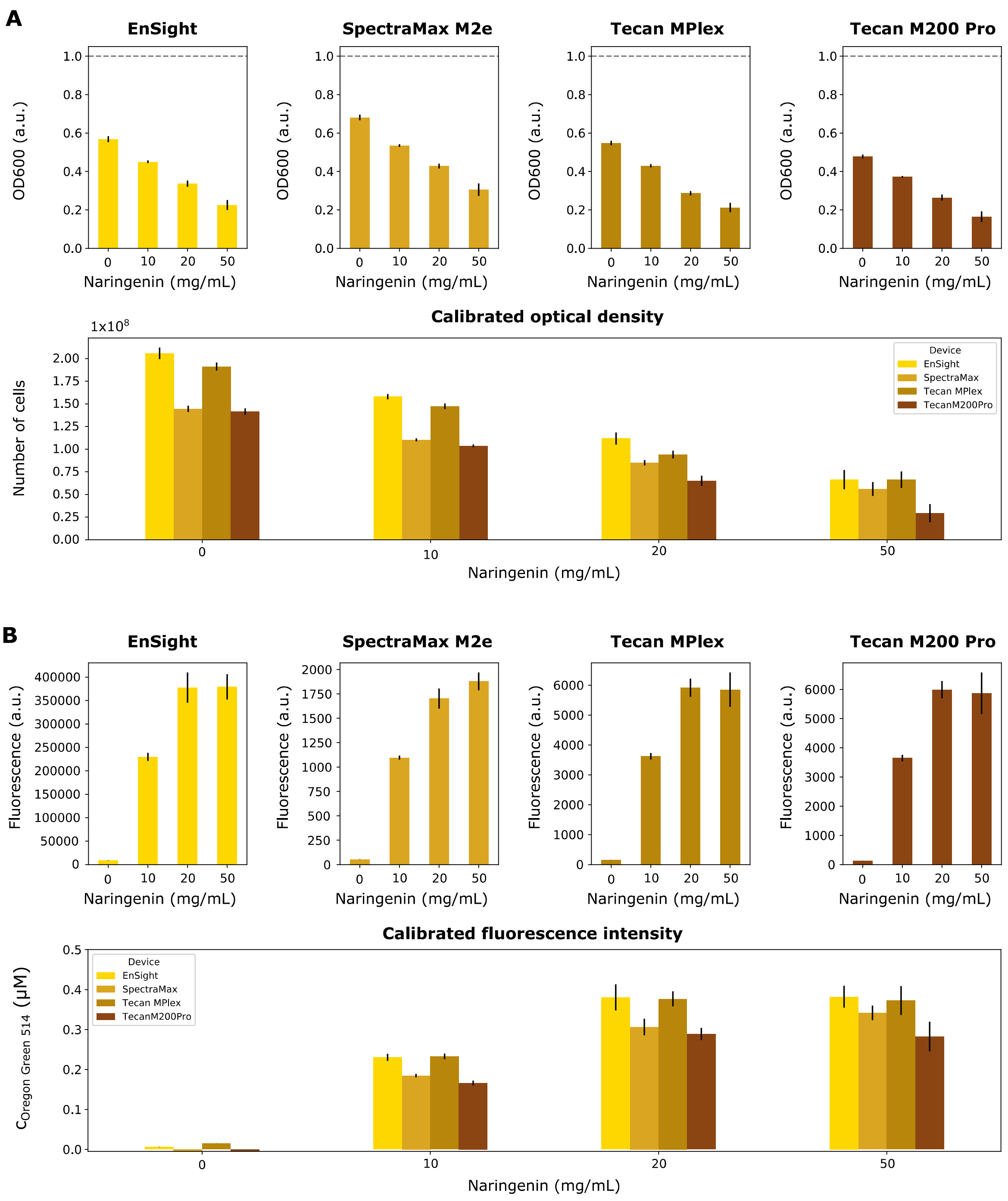

Figure S15: Results of the optical density (A) and fluorescence intensity (B) measurements of the *Escherichia coli* strains expressing the yellow fluorescent protein sYFP2 induced by different naringenin concentrations (0, 10, 20 and 50 mg/L) measured using the different plate readers: EnSight, SpectraMax M2e, Tecan MPlex, Tecan M200 Pro both before and after calibration with the (A) calibration curves for the silica spheres (Figure 4) and (B) calibration curves determined for Oregon Green 514 (Figure S10). Error bars are standard errors and n = 3. Statistical analysis was performed using one-way ANOVA tests followed by post-hoc Tukey test if required (Table S5). OD600 = optical density measured at 600 nm, a.u. = arbitrary units, $c_{\text{Oregon Green 514}}$ = concentration of Oregon Green 514.

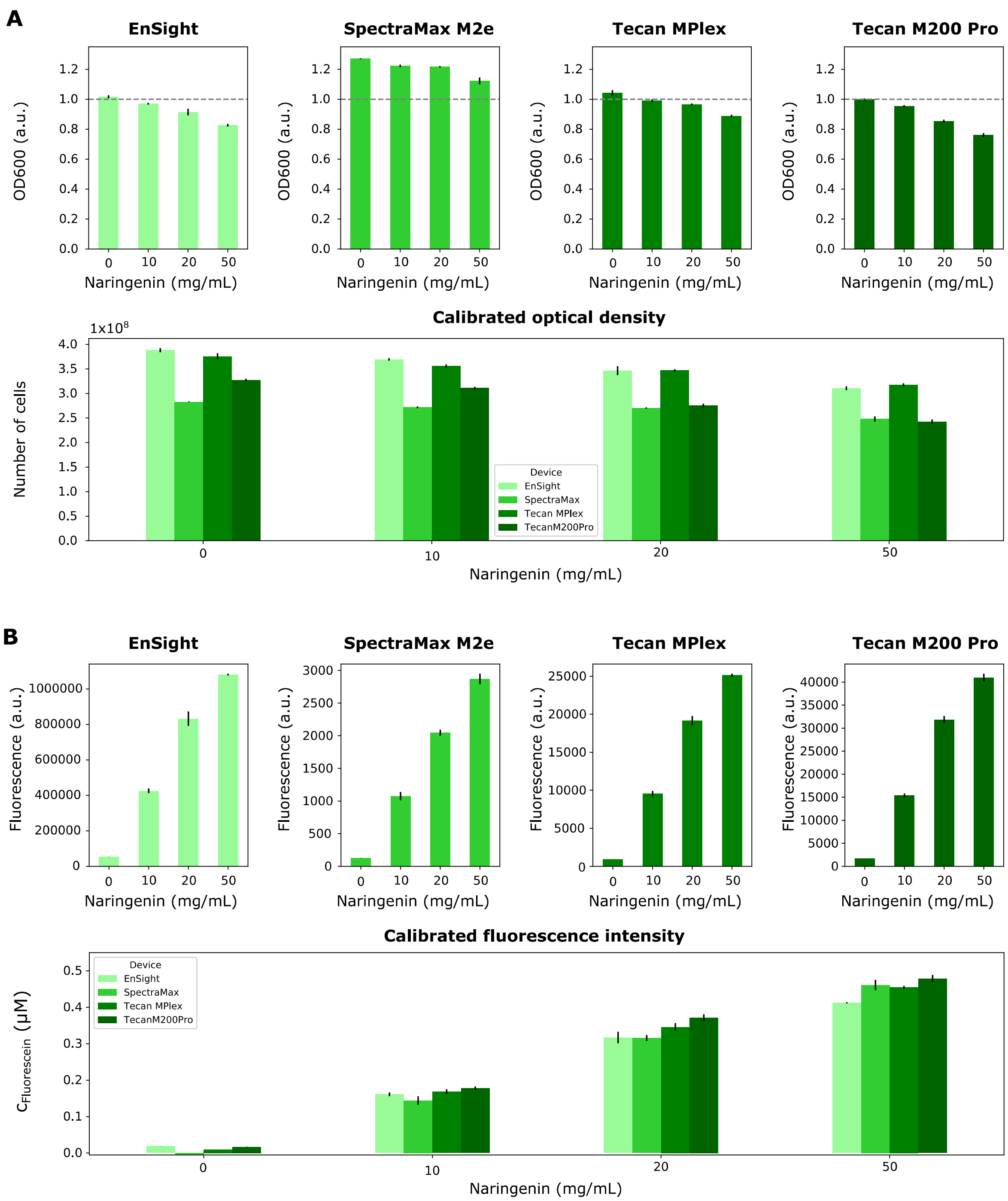

Figure S16: Results of the optical density (A) and fluorescence intensity (B) measurements of the *Escherichia coli* strains expressing the green fluorescent reporter sfGFP induced by different naringenin concentrations (0, 10, 20 and 50 mg/L) measured using the different plate readers: EnSight, SpectraMax M2e, Tecan MPlex, Tecan M200 Pro both before and after calibration with the (A) calibration curves for the silica spheres (Figure 4) and (B) calibration curves determined for fluorescein (Figure S11). Error bars are standard errors and n = 3. Statistical analysis was performed using one-way ANOVA tests followed by post-hoc Tukey test if required (Table S6). OD600 = optical density measured at 600 nm, a.u. = arbitrary units, $c_{Fluorescein}$ = concentration of fluorescein.

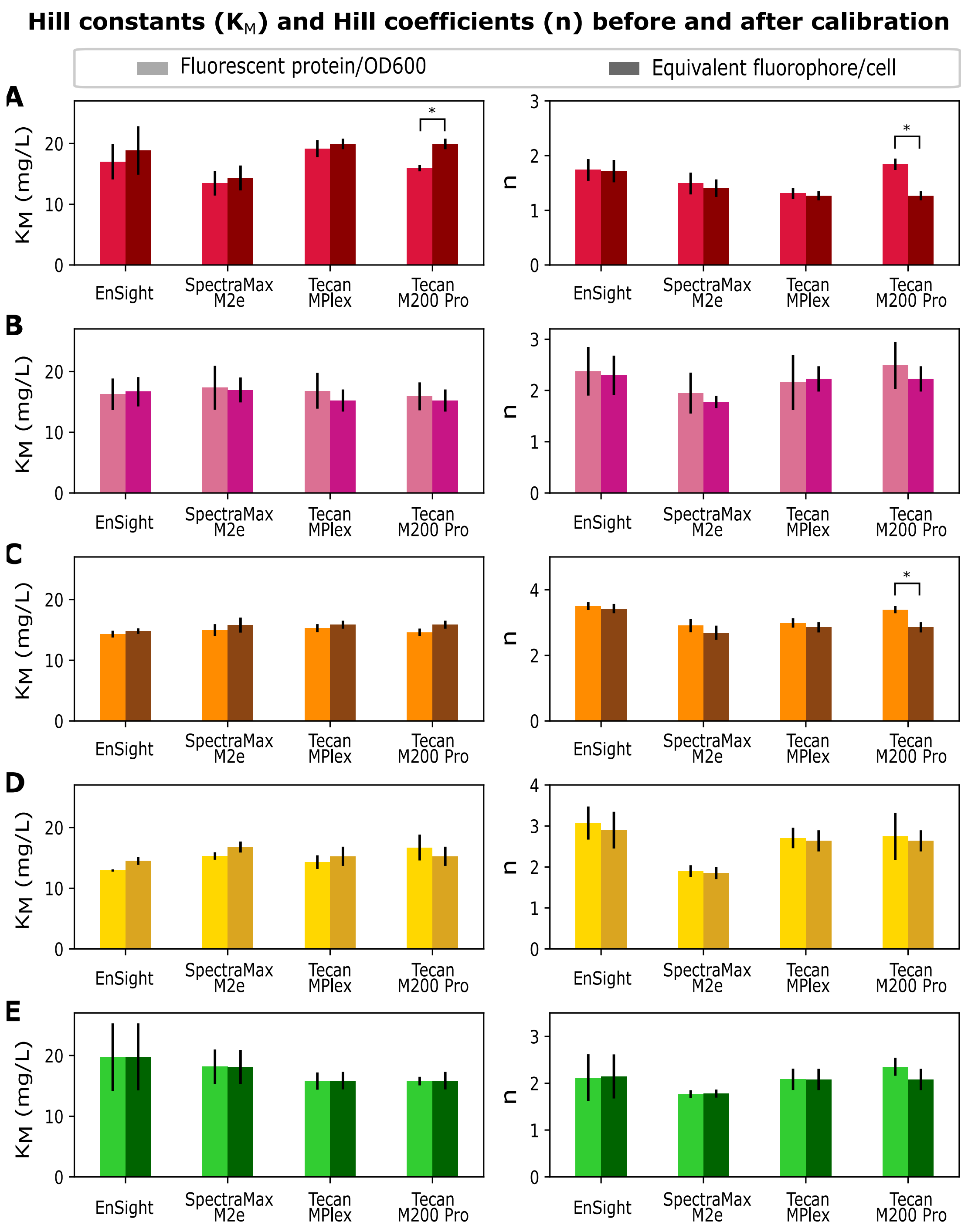

Figure S17: Comparison of the Hill constants (K_M) and Hill coefficients (n), representing respectively the affinity and cooperativity of the transcription factor FdeR and inducer naringenin, between the different plate readers. The results are shown for the different fluorescent reporters before and after calibration: (A) mKate2, (B) mCherry, (C) OFP, (D) sYFP2 and (E) sfGFP. Error bars are standard errors and n = 3. Statistical analysis was performed using one-way ANOVAs testing statistical difference between the different devices and between the parameters obtained before and after calibration. * = p-value < 0.05.

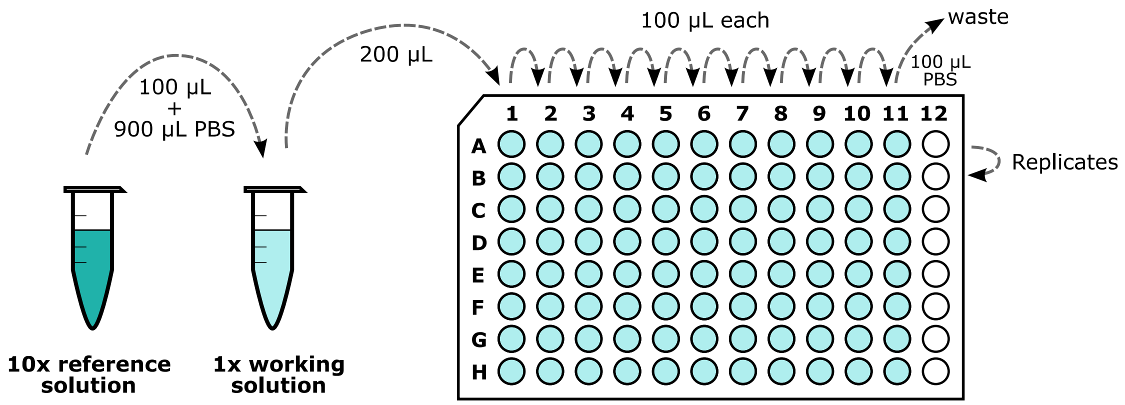

Figure S18: Schematic representation of the protocol used to make the serial dilutions for the standard curves of Texas Red, ROX, TRITC, Oregon Green 514, sodium fluorescein and Pacific Blue. The dilution series were made in octuplicate for each fluorophore. Figure adapted from Beal et al. (2020) .[1]

Table S1: Molar attenuation coefficients (ε) of the different chemical fluorophores calculated using the Beer-Lambert law at the wavelength with maximal absorption (λ_{max}). Quartz cuvettes with a path length of 1 cm were used to carry out the spectrum scans (see Figure S1). The different fluorophore solutions were diluted using either phosphate buffered saline (PBS) solution and/or dimethylsulfoxide (DMSO) to obtain absorbance values within the linear range of the spectrophotometer. TRITC was diluted in PBS instead of DMSO since spectral properties were visibly enhanced upon addition of PBS. c = concentration of the stock solution. Texas Red = sulforhodamine 101 chloride, ROX = carboxy-X-rhodamine, TRITC = tetramethylrhodamine.

Fluorophore	Solvent	λ_{max}	c (mM)	ε ($cm^{-1} \times M^{-1}$)
**Texas Red**	PBS	587	0.007130	68 464.57
**ROX**	PBS	577	0.011231	27 231.43
**TRITC**	PBS	552	0.007305	84 739.52
**Oregon Green 514**	DMSO	507	0.010629	72 798.76
**Fluorescein**	PBS	490	0.010253	56784.51
**Pacific Blue**	DMSO	446	0.014347	56 591.13

Table S2: Results of the statistical analysis performed to compare the calibrated optical density, fluorescence and fluorescence per cell values obtained for the *Escherichia coli* strains expressing mKate2 after performing the standardization protocol between the EnSight, SpectraMax M2e, Tecan M200 Pro and Tecan MPlex plate readers. n = 3. The analysis was performed using a one-way ANOVA test followed by a Tukey's honestly significant difference (HSD) test. The insignificant differences are highlighted using a bold font.

	**0 mg/L Naringenin**	**10 mg/L Naringenin**	**20 mg/L Naringenin**	**50 mg/L Naringenin**
**Calibrated cell counts**	**p-value ANOVA**	**p-value ANOVA**	**p-value ANOVA**	**p-value ANOVA**
	0.0127	0.0229	0.0260	0.0180
**Devices**	**p-value Tukey**	**p-value Tukey**	**p-value Tukey**	**p-value Tukey**
**EnSight - SpectraMax M2e**	0.0134	0.0200	0.1436	0.5013
**EnSight - Tecan MPlex**	0.5286	0.3158	0.9000	0.8213
**EnSight - Tecan M200 Pro**	0.0458	0.0641	0.0403	0.0535
**SpectraMax M2e - Tecan MPlex**	0.0935	0.2625	0.2212	0.1789
**SpectraMax M2e - Tecan M200 Pro**	0.7876	0.8191	0.7855	0.3858
**Tecan MPlex - Tecan M200 Pro**	0.3157	0.6507	0.0630	0.0169
**Equivalent fluorophore concentration**	**p-value ANOVA**	**p-value ANOVA**	**p-value ANOVA**	**p-value ANOVA**
	1.31E-09	0.5161	0.5933	0.8141
**Devices**	**p-value Tukey**	**p-value Tukey**	**p-value Tukey**	**p-value Tukey**
**EnSight - SpectraMax M2e**	0.0010			
**EnSight - Tecan MPlex**	0.0010			
**EnSight - Tecan M200 Pro**	0.0010			
**SpectraMax M2e - Tecan MPlex**	0.0197			
**SpectraMax M2e - Tecan M200 Pro**	0.0010			
**Tecan MPlex - Tecan M200 Pro**	0.0010			
**Equivalent fluorophore concentration/cell**	**p-value ANOVA**	**p-value ANOVA**	**p-value ANOVA**	**p-value ANOVA**
	2.16E-05	0.0078	0.0026	0.0004
**Devices**	**p-value Tukey**	**p-value Tukey**	**p-value Tukey**	**p-value Tukey**
**EnSight - SpectraMax M2e**	0.0010	0.0092	0.0165	0.0172
**EnSight - Tecan MPlex**	0.0037	0.9000	0.8927	0.7176
**EnSight - Tecan M200 Pro**	0.2979	0.3347	0.0268	0.0017
**SpectraMax M2e - Tecan MPlex**	0.0372	0.0140	0.0067	0.0046
**SpectraMax M2e - Tecan M200 Pro**	0.0010	0.1102	0.9000	0.3093
**Tecan MPlex - Tecan M200 Pro**	0.0010	0.4856	0.0106	0.0010

Table S3: Results of the statistical analysis performed to compare the calibrated optical density, fluorescence and fluorescence per cell values obtained for the *Escherichia coli* strains expressing mCherry after performing the standardization protocol between the EnSight, SpectraMax M2e, Tecan M200 Pro and Tecan MPlex plate readers. n = 3. The analysis was performed using a one-way ANOVA test followed by a Tukey's honestly significant difference (HSD) test. The insignificant differences are highlighted using a bold font.

	**0 mg/L Naringenin**	**10 mg/L Naringenin**	**20 mg/L Naringenin**	**50 mg/L Naringenin**
**Calibrated cell counts**	**p-value ANOVA**	**p-value ANOVA**	**p-value ANOVA**	**p-value ANOVA**
	0.0304	0.0136	0.0221	0.0420
**Devices**	**p-value Tukey**	**p-value Tukey**	**p-value Tukey**	**p-value Tukey**
**EnSight - SpectraMax M2e**	0.1325	0.1715	0.7184	0.8374
**EnSight - Tecan MPlex**	0.7021	0.2995	0.1188	0.1461
**EnSight - Tecan M200 Pro**	0.4836	0.5160	0.7804	0.9000
**SpectraMax M2e - Tecan MPlex**	0.0297	0.0123	0.0278	0.0473
**SpectraMax M2e - Tecan M200 Pro**	0.7279	0.7905	0.9000	0.9000
**Tecan MPlex - Tecan M200 Pro**	0.1236	0.0417	0.0329	0.0690
**Equivalent fluorophore concentration**	**p-value ANOVA**	**p-value ANOVA**	**p-value ANOVA**	**p-value ANOVA**
	9.75E-17	1.03E-05	0.2178	0.4862
**Devices**	**p-value Tukey**	**p-value Tukey**	**p-value Tukey**	**p-value Tukey**
**EnSight - SpectraMax M2e**	0.0010	0.0039		
**EnSight - Tecan MPlex**	0.0010	0.0019		
**EnSight - Tecan M200 Pro**	0.0010	0.0023		
**SpectraMax M2e - Tecan MPlex**	0.0192	0.9000		
**SpectraMax M2e - Tecan M200 Pro**	0.0010	0.0010		
**Tecan MPlex - Tecan M200 Pro**	0.0010	0.0010		
**Equivalent fluorophore concentration/cell**	**p-value ANOVA**	**p-value ANOVA**	**p-value ANOVA**	**p-value ANOVA**
	7.26E-07	9.84E-07	0.0190	0.0045
**Devices**	**p-value Tukey**	**p-value Tukey**	**p-value Tukey**	**p-value Tukey**
**EnSight - SpectraMax M2e**	0.0010	0.0010	0.0423	0.0170
**EnSight - Tecan MPlex**	0.0039	0.0241	0.9000	0.6273
**EnSight - Tecan M200 Pro**	0.0010	0.0020	0.9000	0.9000
**SpectraMax M2e - Tecan MPlex**	0.0060	0.0010	0.0385	0.0036
**SpectraMax M2e - Tecan M200 Pro**	0.0010	0.0010	0.0265	0.0371
**Tecan MPlex - Tecan M200 Pro**	0.0010	0.0010	0.9000	0.3476

Table S4: Results of the statistical analysis performed to compare the calibrated optical density, fluorescence and fluorescence per cell values obtained for the Escherichia coli strains expressing OFP after performing the standardization protocol between the EnSight, SpectraMax M2e, Tecan M200 Pro and Tecan MPlex plate readers. n = 3. The analysis was performed using a one-way ANOVA test followed by a Tukey's honestly significant difference (HSD) test. The insignificant differences are highlighted using a bold font.

	**0 mg/L Naringenin**	**10 mg/L Naringenin**	**20 mg/L Naringenin**	**50 mg/L Naringenin**
**Calibrated cell counts**	**p-value ANOVA**	**p-value ANOVA**	**p-value ANOVA**	**p-value ANOVA**
	3.12E-07	2.74E-08	3.86E-06	0.0003
**Devices**	**p-value Tukey**	**p-value Tukey**	**p-value Tukey**	**p-value Tukey**
**EnSight - SpectraMax M2e**	0.0010	0.0010	0.0010	0.0094
**EnSight - Tecan MPlex**	0.0261	0.0086	0.1464	0.6786
**EnSight - Tecan M200 Pro**	0.0010	0.0010	0.0010	0.0019
**SpectraMax M2e - Tecan MPlex**	0.0010	0.0010	0.0010	0.0024
**SpectraMax M2e - Tecan M200 Pro**	0.0010	0.0010	0.5299	0.5682
**Tecan MPlex - Tecan M200 Pro**	0.0010	0.0010	0.0010	0.0010
**Equivalent fluorophore concentration**	**p-value ANOVA**	**p-value ANOVA**	**p-value ANOVA**	**p-value ANOVA**
	7.65E-16	0.0001	0.0021	6.63E-05
**Devices**	**p-value Tukey**	**p-value Tukey**	**p-value Tukey**	**p-value Tukey**
**EnSight - SpectraMax M2e**	0.0010	0.0013	0.0015	0.0010
**EnSight - Tecan MPlex**	0.4419	0.8318	0.3173	0.0095
**EnSight - Tecan M200 Pro**	0.0010	0.0565	0.2706	0.0053
**SpectraMax M2e - Tecan MPlex**	0.0010	0.0033	0.0144	0.0021
**SpectraMax M2e - Tecan M200 Pro**	0.0010	0.0010	0.0171	0.0036
**Tecan MPlex - Tecan M200 Pro**	0.0010	0.0183	0.9000	0.9000
**Equivalent fluorophore concentration/cell**	**p-value ANOVA**	**p-value ANOVA**	**p-value ANOVA**	**p-value ANOVA**
	2.00E-13	4.14E-06	3.16E-06	3.98E-07
**Devices**	**p-value Tukey**	**p-value Tukey**	**p-value Tukey**	**p-value Tukey**
**EnSight - SpectraMax M2e**	0.0010	0.0010	0.0010	0.0010
**EnSight - Tecan MPlex**	0.0177	0.5493	0.6594	0.0416
**EnSight - Tecan M200 Pro**	0.0010	0.5455	0.0010	0.0010
**SpectraMax M2e - Tecan MPlex**	0.0010	0.0010	0.0010	0.0010
**SpectraMax M2e - Tecan M200 Pro**	0.0010	0.0010	0.0056	0.0149
**Tecan MPlex - Tecan M200 Pro**	0.0010	0.0963	0.0010	0.0010

Table S5: Results of the statistical analysis performed to compare the calibrated optical density, fluorescence and fluorescence per cell values obtained for the *Escherichia coli* strains expressing sYFP2 after performing the standardization protocol between the EnSight, SpectraMax M2e, Tecan M200 Pro and Tecan MPlex plate readers. n = 3. The analysis was performed using a one-way ANOVA test followed by a Tukey's honestly significant difference (HSD) test. The insignificant differences are highlighted using a bold font.

	**0 mg/L Naringenin**	**10 mg/L Naringenin**	**20 mg/L Naringenin**	**50 mg/L Naringenin**
**Calibrated cell counts**	**p-value ANOVA**	**p-value ANOVA**	**p-value ANOVA**	**p-value ANOVA**
	1.56E-05	5.57E-07	0.0013	0.0719
**Devices**	**p-value Tukey**	**p-value Tukey**	**p-value Tukey**	**p-value Tukey**
**EnSight - SpectraMax M2e**	0.0010	0.0010	0.0247	
**EnSight - Tecan MPlex**	0.0626	0.0626	0.1437	
**EnSight - Tecan M200 Pro**	0.0010	0.0010	0.0010	
**SpectraMax M2e - Tecan MPlex**	0.0010	0.0010	0.6050	
**SpectraMax M2e - Tecan M200 Pro**	0.3031	0.3031	0.0934	
**Tecan MPlex - Tecan M200 Pro**	0.0010	0.0010	0.0163	
**Equivalent fluorophore concentration**	**p-value ANOVA**	**p-value ANOVA**	**p-value ANOVA**	**p-value ANOVA**
	8.11E-13	0.0002	0.0452	0.1746
**Devices**	**p-value Tukey**	**p-value Tukey**	**p-value Tukey**	**p-value Tukey**
**EnSight - SpectraMax M2e**	0.0010	0.0060	0.1820	
**EnSight - Tecan MPlex**	0.0010	0.9000	0.9000	
**EnSight - Tecan M200 Pro**	0.0010	0.0010	0.0856	
**SpectraMax M2e - Tecan MPlex**	0.0010	0.0044	0.2121	
**SpectraMax M2e - Tecan M200 Pro**	0.0010	0.2896	0.9000	
**Tecan MPlex - Tecan M200 Pro**	0.0010	0.0010	0.1004	
**Equivalent fluorophore concentration/cell**	**p-value ANOVA**	**p-value ANOVA**	**p-value ANOVA**	**p-value ANOVA**
	1.89E-10	0.0245	0.3310	0.0578
**Devices**	**p-value Tukey**	**p-value Tukey**	**p-value Tukey**	**p-value Tukey**
**EnSight - SpectraMax M2e**	0.0010	0.0174		
**EnSight - Tecan MPlex**	0.0010	0.1935		
**EnSight - Tecan M200 Pro**	0.0010	0.1057		
**SpectraMax M2e - Tecan MPlex**	0.0010	0.3693		
**SpectraMax M2e - Tecan M200 Pro**	0.0010	0.5850		
**Tecan MPlex - Tecan M200 Pro**	0.0010	0.9000		

Table S6: Results of the statistical analysis performed to compare the calibrated optical density, fluorescence and fluorescence per cell values obtained for the *Escherichia coli* strains expressing sfGFP after performing the standardization protocol between the EnSight, SpectraMax M2e, Tecan M200 Pro and Tecan MPlex plate readers. n = 3. The analysis was performed using a one-way ANOVA test followed by a Tukey's honestly significant difference (HSD) test. The insignificant differences are highlighted using a bold font.

	0 mg/L Naringenin	10 mg/L Naringenin	20 mg/L Naringenin	50 mg/L Naringenin
**Calibrated cell counts**	**p-value ANOVA**	**p-value ANOVA**	**p-value ANOVA**	**p-value ANOVA**
	2.73E-07	1.89E-08	3.94E-06	2.12E-06
**Devices**	**p-value Tukey**	**p-value Tukey**	**p-value Tukey**	**p-value Tukey**
**EnSight - SpectraMax M2e**	0.001	0.001	0.001	0.001
**EnSight - Tecan MPlex**	0.1969	0.0316	0.9	0.6728
**EnSight - Tecan M200 Pro**	0.001	0.001	0.001	0.001
**SpectraMax M2e - Tecan MPlex**	0.001	0.001	0.001	0.001
**SpectraMax M2e - Tecan M200 Pro**	0.001	0.001	0.8646	0.775
**Tecan MPlex - Tecan M200 Pro**	0.001	0.001	0.001	0.001
**Equivalent fluorophore concentration**	**p-value ANOVA**	**p-value ANOVA**	**p-value ANOVA**	**p-value ANOVA**
	4.19E-13	0.0629	0.0256	0.0049
**Devices**	**p-value Tukey**	**p-value Tukey**	**p-value Tukey**	**p-value Tukey**
**EnSight - SpectraMax M2e**	0.001		0.9	0.0214
**EnSight - Tecan MPlex**	0.001		0.3565	0.0412
**EnSight - Tecan M200 Pro**	0.0208		0.04	0.0037
**SpectraMax M2e - Tecan MPlex**	0.001		0.3232	0.9
**SpectraMax M2e - Tecan M200 Pro**	0.001		0.0358	0.5482
**Tecan MPlex - Tecan M200 Pro**	0.001		0.4309	0.3233
**Equivalent fluorophore concentration/cell**	**p-value ANOVA**	**p-value ANOVA**	**p-value ANOVA**	**p-value ANOVA**
	4.29E-13	1.80E-02	9.52E-04	3.74E-05
**Devices**	**p-value Tukey**	**p-value Tukey**	**p-value Tukey**	**p-value Tukey**
**EnSight - SpectraMax M2e**	0.001	0.0938	0.0253	0.001
**EnSight - Tecan MPlex**	0.001	0.6846	0.6547	0.4905
**EnSight - Tecan M200 Pro**	0.1073	0.0176	0.001	0.001
**SpectraMax M2e - Tecan MPlex**	0.001	0.4042	0.1284	0.0014
**SpectraMax M2e - Tecan M200 Pro**	0.001	0.6337	0.1044	0.4492
**Tecan MPlex - Tecan M200 Pro**	0.001	0.0815	0.0036	0.001

Table S7: Results of the statistical analysis performed to compare the calibrated optical density, fluorescence and fluorescence per cell values obtained for the *Escherichia coli* strains expressing mTagBFP after performing the standardization protocol between the EnSight, SpectraMax M2e, Tecan M200 Pro and Tecan MPlex plate readers. n = 3. The analysis was performed using a one-way ANOVA test followed by a Tukey's honestly significant difference (HSD) test. The insignificant differences are highlighted using a bold font.

**Devices**	**p-value Tukey**	**p-value Tukey**	**p-value Tukey**	**p-value Tukey**
**EnSight - SpectraMax M2e**	0.0010	0.0010	0.0010	0.0010
**EnSight - Tecan MPlex**	0.6042	0.0155	0.9000	0.4255
**EnSight - Tecan M200 Pro**	0.0010	0.0010	0.0011	0.0010
**SpectraMax M2e - Tecan MPlex**	0.0010	0.0010	0.0010	0.0010
**SpectraMax M2e - Tecan M200 Pro**	0.0078	0.0010	0.9000	0.3816
**Tecan MPlex - Tecan M200 Pro**	0.0024	0.0010	0.0016	0.0010
**Equivalent fluorophore concentration**	**p-value ANOVA**	**p-value ANOVA**	**p-value ANOVA**	**p-value ANOVA**
	2.70E-14	2.87E-06	6.58E-05	4.25E-03
**Devices**	**p-value Tukey**	**p-value Tukey**	**p-value Tukey**	**p-value Tukey**
**EnSight - SpectraMax M2e**	0.0010	0.0010	0.7670	0.8067
**EnSight - Tecan MPlex**	0.0010	0.0231	0.0979	0.7929
**EnSight - Tecan M200 Pro**	0.0010	0.0010	0.0010	0.0119
**SpectraMax M2e - Tecan MPlex**	0.0010	0.0069	0.3455	0.3610
**SpectraMax M2e - Tecan M200 Pro**	0.0010	0.0010	0.0010	0.0382
**Tecan MPlex - Tecan M200 Pro**	0.0367	0.0010	0.0010	0.0039
**Equivalent fluorophore concentration/cell**	**p-value ANOVA**	**p-value ANOVA**	**p-value ANOVA**	**p-value ANOVA**
	1.92E-12	2.50E-06	8.66E-08	2.04E-05
**Devices**	**p-value Tukey**	**p-value Tukey**	**p-value Tukey**	**p-value Tukey**
**EnSight - SpectraMax M2e**	0.0010	0.0318	0.0012	0.0104
**EnSight - Tecan MPlex**	0.0033	0.1168	0.0338	0.6103
**EnSight - Tecan M200 Pro**	0.0010	0.0010	0.0010	0.0010
**SpectraMax M2e - Tecan MPlex**	0.0010	0.7747	0.0010	0.0023
**SpectraMax M2e - Tecan M200 Pro**	0.0010	0.0010	0.0010	0.0042
**Tecan MPlex - Tecan M200 Pro**	0.0777	0.0010	0.0010	0.0010

Table S8: Oligonucleotides used during the development of the different strains used for the standardization of plate reader experiments.

Name	Sequence (5' → 3')
**oMEMO9543_PfdeA-FW**	TAGCCCATGGTTAGGCGCTGCTCTCCGTTGTTGTGCTT GTTC
**oMEMO9544_PfdeA-RV**	CAGCGCCTAACCATGGGCTAGCATTGCCGTTCCGACTG ATTG
**oMEMO9451_AarI-FW**	ACGTGGTCTCGAAGGCGTAGCAGGTGACGACGTACGTC GTAG
**oMEMO9452_AarI-RV**	ACGTGGTCTCCACTCTATGATGTGCAGGTGACGTTACG AGGTCCTACGACGTACGTCGTCACC
**oMEMO9650_mCherry-FW**	TAGGAGACAAGGAGACAGACATGGTGAGCAAGGGCGAG GAG
**oMEMO9651_mCherry-RV**	AAACCGCTAGCCCATGGTTATTACTTGTACAGCTCGTC CATGCCGCCGGTG
**oMEMO9652_OFP-FW**	TAGGAGACAAGGAGACAGACATGAACCTGTCCAAAAAC GTATC
**oMEMO9653_OFP-RV**	AAACCGCTAGCCCATGGTTATTATTTTGGCAGATCGCT AACG
**oMEMO9654_sYFP2-FW**	TAGGAGACAAGGAGACAGACATGGTTAGCAAGGGCGAA GAAC
**oMEMO9655_sYFP2-RV**	AAACCGCTAGCCCATGGTTATTATTTATACAGCTCATC CATACC
**oMEMO9656_sfGFP-FW**	TAGGAGACAAGGAGACAGACATGGGCAAGGGCGAAGAG CTTTTTACCG
**oMEMO9657_sfGFP-RV**	AAACCGCTAGCCCATGGTTATTACTTATAGAGTTCATC CATGCCATGAG
**oMEMO9660_mTagBFP-FW**	TAGGAGACAAGGAGACAGACATGAGCGAACTGATCAAA GAG
**oMEMO9661_mTagBFP-RV**	AAACCGCTAGCCCATGGTTATTAATTCAGTTTATGACC CAGCTTGCTAGG
**oMEMO9662_pBBR1MCS2-FW**	TAACCATGGGCTAGCGGTTTG
**oMEMO9663_pBBR1MCS2-RV**	GTCTGTCTCCTTGTCTCCTAC

(1) Beal, J.; Farny, N. G.; Haddock-Angelli, T.; Selvarajah, V.; Baldwin, G. S.; Buckley-Taylor, R.; Gershater, M.; Kiga, D.; Marken, J.; Sanchania, V.; Sison, A.; Workman, C. T. Robust Estimation of Bacterial Cell Count from Optical Density. *Nat. Commun. Biol.* **2020**, *3* (1), 512.

Standardization of fluorescence plate reader assays across the visible light spectrum

Goal:

Standardization of fluorescence reporter assays performed on plate readers by converting the arbitrary units (a.u.) generated by the device into standardized units (concentration of a chemical fluorophore per cell). Calibration of optical density measurements for *E. coli* is done using silica spheres with a comparable size and scatter to *E. coli*. The following chemical fluorophores were selected for the calibration of a range of fluorescent proteins across the visible light spectrum:

**Fluorophore**	**Fluorescent reporter**
Pacific blue	mTagBFP
Sodium fluorescein	sfGFP
Oregon Green 514	sYFP2
TRITC	OFP
Texas Red	mCherry
Texas Red	mKate2

Calibration curves are specific for each device and must be determined again when using a new plate reader.

Materials:

- Black 96 well plates
- Transparent 96 well plates
- Volumetric flasks (50, 100 and 200 mL)
- Weighing boats
- 1.5 mL Eppendorf tubes (amber or shielded with foil)
- dH_2O
- PBS
- DMSO
- Silica spheres (beads) with diameter of 1.0 µm (for *E. coli*):

Note: freezing of the beads might cause irreversible aggregation!

**Product**	**Supplier**	**Product nr.**
Silica spheres	Polysciences	25343-1.5 Silica Microspheres - Dry, 1.0µm (1,5g)

- Chemical fluorophores:

**Product**	**Supplier**	**Product nr.**
Pacific Blue	Gentaur (AAT bioquest)	1015693484 - 570 PacBlue succinimidyl ester (5 mg)
Sodium fluorescein	Sigma	30181-100MG Fluorescein sodium salt, analytical standard
TRITC	Sigma	87918-10MG Tetramethylrhodamine isothiocyanate mixed isomers
Oregon Green 514	Fisher	O6139 Oregon Green™ 514 Carboxylic Acid, Succinimidyl Ester
Texas Red	Sigma	S3388-5MG - Sulforhodamine 101 acid chloride

Equipment:

- Plate reader with the appropriate optical density and fluorescence measurement properties
- Incubator
- Analytical scales

Procedure:

Silica bead calibration:

1. Prepare a stock solution containing $2x10^6$ spheres by dissolving 0.5 g of the silica spheres with a diameter of 1.05 µm in 206.226 mL dH_2O
2. Stir the solution for at least two hours to ensure complete dissolution of the spheres

   Note: the sphere solution must be stirred immediately before use since the beads sediment rapidly!
3. Prepare the calibration plate by making a dilution series of the silica spheres in dH_2O in a transparent 96 well plate (columns 1-12). Eight replicates of this dilution series are made (rows A-H).

**Dilution**	1	2	3	4	5	6	7	8	9	10	11	12
**Sphere stock solution (µL)**	150	135	120	105	90	75	60	45	30	15	7.5	0
**dH_2O (µL)**	0	15	30	45	60	75	90	105	120	135	142.5	150

4. Measure the optical density at 600 nm (OD600) of the dilution series using a plate reader

   Note: resuspend the silica spheres in the dH_2O immediately before measuring the OD600 by pipetting up and down.

5. Determine the correlation between the optical density and the silica sphere concentration using linear regression. Remove all data points with an OD600 > 1 since these are in the multiple scattering regime.

Fluorophore calibration:

Safety note: wear gloves since the fluorophores are known to have carcinogenic properties!
Note: due to the low stability of the fluorophore solutions, the solutions must be made on the day that the calibration experiments will be performed.

1. Prepare the stock solution for the required fluorophore using the appropriate solvent:

**Fluorophore**	**Solvent**	**$c_{stock\ solution}$ (mM)**	**MW (g/mol)**	**$m_{fluorophore}$ (mg)**	**$V_{solvent}$ (mL)**
Pacific blue	DMSO	0.1	339.2086	1.696	50
Sodium fluorescein	PBS	1	332.31	75.28	200
Oregon Green 514	DMSO	0.1	609.4341	3.0471	50
TRITC	DMSO	0.1	443.52	2.2176	50
Texas Red	PBS	0.1	625.2	6.2	100

   a. Weigh the fluorophore using an analytical scale. Try to reach the target weight as close as possible. Write down the actual mass of fluorophore that will be added.

b. Dissolve the fluorophore in the appropriate volume of solvent using a volumetric flask. Remove all the fluorophore leftover in the weighing boat by adding a small volume of the solvent while preparing the solution.

c. Determine the actual concentration of the stock solution using the mass of the fluorophore that was added.

Fluorophore	Solvent	$C_{10x\ reference\ solution}$ (μM)
Pacific blue	DMSO	500
Sodium fluorescein	PBS	100
Oregon Green 514	DMSO	100
TRITC	DMSO	100
Texas Red	PBS	100

2. Prepare 10x reference solutions by diluting the stock solutions in their respective solvents. Start from the actual concentration of the stock solution.

3. Prepare 1x working solutions by mixing 100 μL of the 10x reference solution with 900 μL PBS.

Note: also fluorophores dissolved in DMSO should from this point forward be diluted using PBS.

4. Prepare the dilution series of the chemical fluorophore in a black 96 well plate. The dilutions are made in columns 1-12 and replicates are made in rows A-H.

a. Add 100 μL of PBS to the wells in columns 2-12.

b. Add 200 μL of the fluorophore 1x working solution to the wells of column 1.

c. Make the dilution series by transferring 100 μL of column 1 to row 2. Mix well by pipetting up and down. Repeat this step until column 11. Discard 100 μL of the diluted fluorophore solution. Column 12 contains only PBS!

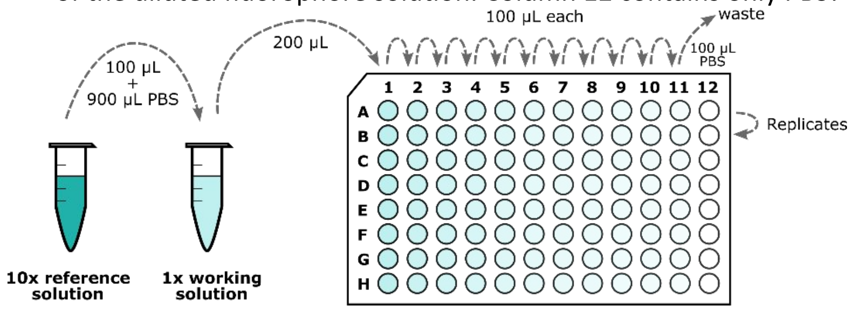

5. Measure the fluorescence of the dilution series using the appropriate excitation and emission wavelengths:

Fluorophore	Fluorescent reporter	Excitation wavelength (nm)	Emission wavelength (nm)
Pacific blue	mTagBFP	399	456
Sodium fluorescein	sfGFP	480	520
Oregon Green 514	sYFP2	515	545
TRITC	OFP	543	573
Texas Red	mCherry	580	610
Texas Red	mKate2	588	633

6. Determine the correlation between the fluorophore concentration and the arbitrary units generated by the plate reader using linear regression. Remove all data points exceeding the saturation limit or linear range of the plate reader. To maximize

compatibility with fluorescence reporter assays, you should measure calibration curves with a range of gain settings if your machine has this as a setting. When measuring cell-based fluorescence measurements, gain settings should be limited to one of these pre-calibrated settings.

Standardization of the plate reader assay

1. Transform the arbitrary units generated during OD600 measurement of your growth experiment using the silica sphere calibration curve set up for your device.
2. Transform the arbitrary units generated during fluorescence measurements of your fluorescent reporter using the chemical fluorophore calibration curve. Note that it is essential that the fluorescence of the fluorescent reporter is measured at the same wavelengths and gain setting that are used for the chemical fluorophore.
3. Normalize the obtained chemical fluorophore concentration per well for the number of cells in the well to obtain the concentration of equivalent chemical fluorophore per cell.
